# Efficacy of Exercise on Severity of Paclitaxel-Induced Peripheral Neuropathy and Improving Quality of Life in Women with Non-metastatic Breast Cancer: Results of an Interim Analysis from an Ongoing Randomized Clinical Trial (ExPIN Trial)

**DOI:** 10.1177/15347354251398002

**Published:** 2025-12-18

**Authors:** Mangaiyarkarasi Sekaran, Ananth Pai, Arvind N. Prabhu, Vasudeva Bhat K, Chethan Channaveera, Jeffrey Pradeep Raj, Vijetha Shenoy Belle, Sharada Mailankody, Karthik S. Udupa

**Affiliations:** 1 Department of Medical Oncology, Kasturba Medical College, Manipal, Manipal Academy of Higher Education, Manipal, Karnataka, India; 2 Department of Neurology, Kasturba Medical College, Manipal, Manipal Academy of Higher Education, Manipal, Karnataka, India; 3 Department of Paediatric Oncology, Kasturba Medical College, Manipal, Manipal Academy of Higher Education, Manipal, Karnataka, India; 4 Department of Physical Medicine and Rehabilitation, Atal Bihari Vajpayee Institute of Medical Sciences and Dr. Ram Manohar Lohia Hospital, New Delhi, India; 5 Division of Clinical Pharmacology, Kasturba Medical College, Manipal, Manipal Academy of Higher Education, Manipal, Karnataka, India; 6 Department of Biochemistry, Kasturba Medical College, Manipal, Manipal Academy of Higher Education, Manipal, Karnataka, India

**Keywords:** exercise, paclitaxel, peripheral neuropathy, breast cancer therapy, quality of life, nerve conduction studies

## Abstract

**Background:**

Paclitaxel-induced peripheral neuropathy (PIPN) is a dose-limiting adverse effect during paclitaxel therapy, with no effective treatment and often incomplete recovery. This study assessed the effects of exercise on PIPN severity in newly diagnosed advanced breast cancer patients.

**Methods:**

The ExPIN Trial is an ongoing randomized clinical trial. This interim analysis, conducted between November 2022 and December 2023, included adult patients with locally advanced, non-metastatic breast cancer. Participants were randomized into interventional and control groups for 0 to 12 weeks. The primary outcome was nerve conduction studies. Secondary outcomes included balance scores, taxane-induced toxicity-related quality-of-life scores, emotional functioning, physical functioning, and functional impairments assessed at baseline, after chemotherapy, and at the first follow-up.

**Results:**

Seventy patients were randomized. The mean (SD) age was 49.46 (9.52) years in the interventional group and 49.09 (8.10) years in the control group. At the completion of paclitaxel therapy, the amplitudes of the ulnar nerve (estimate: 1.98, *P* = .007), common peroneal nerve (estimate: 1.88, *P* = .003), and sural nerve (estimate: 6.20, *P* < .001) were significantly higher in the interventional group. On the EORTC scale (lower scores indicate better quality of life), sensory and motor scores significantly decreased in the interventional group by the end of therapy (−5.46 and −3.82, *P* < .001), indicating improvement. These improvements were sustained at follow-up (−1.99, *P* = .001, and −4.20, *P* < .001, respectively).

**Conclusions:**

Exercise intervention during paclitaxel therapy significantly reduced PIPN severity and improved quality of life. Findings are preliminary and warrant further confirmation.

## Introduction

Long-term neurotoxicity is a significant challenge for cancer survivors, particularly those treated for breast cancer. Among the most troubling side effects is chemotherapy-induced peripheral neuropathy (CIPN), which can disrupt treatment by requiring dose reductions or even early discontinuation of chemotherapy. Neurotoxicity is a well-known complication of chemotherapy, particularly taxanes such as paclitaxel, which commonly leads to peripheral neuropathy. Symptoms such as burning sensations in the hands and feet, along with loss of reflexes, can persist in 11% to over 80% of patients, depending on the individual and the treatment regimen.^1^

Paclitaxel-induced neuropathy predominantly affects the sensory nerve fibers. Approximately 40% of patients receiving taxane-baseded chemotherapy experience neuropathy within 2 years of starting treatment.^2^ This condition, which can be painful and interfere with activities of daily living, is often managed with duloxetine, a medication that has been effective in reducing symptoms. However, other medications, such as gabapentin and tricyclic antidepressants, have shown limited benefits. The severity of neuropathy caused by taxanes, such as paclitaxel and docetaxel, tends to be dose- and schedule-dependent. Up to 30% of patients develop moderate-to-severe neuropathy, with 5% to 10% experiencing grade 3 or 4 symptoms.^3^ Early identification and prompt intervention can prevent the condition from worsening, but in severe cases, recovery may be incomplete even after stopping treatment. Paclitaxel can also cause an acute pain syndrome, often targeting the legs, hips, and back, which emerges within days of treatment and tends to worsen with subsequent doses.^4^

Nerve conduction studies provide insight into the extent of damage, often revealing reduced or absent sensory nerve action potentials (SNAP), especially in the sural nerve. These findings indicate axonal loss in sensory nerves.^5^ Risk factors for severe neuropathy include pre-existing nerve damage, longer infusion durations, and platinum-based combination therapies. Weekly paclitaxel regimens have been shown to result in higher neurotoxicity rates compared to less frequent schedules. For example, 1 trial reported grade 3 neuropathy in 24% of patients on a weekly schedule versus 12% on a 3-weekly schedule.^3,6,7^

Exercise has emerged as a promising approach to manage CIPN. Research suggests it can reduce symptoms, improve balance, and enhance the quality of life for breast cancer survivors. However, the relationship between exercise and its effectiveness in managing CIPN remains unclear.^8,9^ The ExPIN trial (Exercise for Paclitaxel-Induced Neuropathy) therefore, aims to address this gap by evaluating how exercise interventions can reduce the severity of CIPN and improve the overall quality of life in breast cancer patients undergoing weekly paclitaxel therapy.

## Material and Methods

### Ethics

The institutional ethics committee approved the study vide study reference number: 161/2022 dated 16/11/2022. A written informed consent was obtained from all potential participants before initiating any study related procedure and screening for eligibility. The study was also registered prospectively in the Clinical Trials Registry of India. (CTRI/2022/10/046907) and was conducted in accordance with the Indian rules and regulations that are backed by the Declaration of Helsinki (2013).

### Study Design and Setting

It is an ongoing open label, parallel group, randomized-controlled trial (RCT) conducted in a tertiary care teaching hospital located in coastal Karnataka state, India. We present the results of the apriori planned interim analysis from an ongoing RCT corresponding to the time period between 2022 and 2023.

### Participants

Women aged 18 years and above, diagnosed for the first time with histologically confirmed, non-metastatic cancer (stage I-III), and scheduled to receive weekly paclitaxel chemotherapy at a dose of 80 mg/m^2^ were included in the study. Patients were excluded if they had a history of previously diagnosed or undiagnosed peripheral neuropathy, an absence of SNAP or Compound Muscle Action Potential (CMAP) in baseline (T0) nerve conduction studies, severe ischemic heart disease (ejection fraction < 30% or symptoms of breathlessness), severe congestive cardiac failure, acute myocardial infarction within the past 3 months, or acute exacerbations of chronic obstructive pulmonary disease (COPD) or asthma. Additionally, individuals with a history of moderate to severe alcohol use disorder (as per the Diagnostic and Statistical Manual of Mental Disorders [DSM]-5 criteria) or those unwilling to provide consent for participation were excluded

### Interventions

Participants from the intervention group underwent a targeted exercise program based on sensory activation (tactile stimulation by rubbing the hands and feet with mild-cotton, different textures, moderate-sandpaper, velcro and advanced -submerge with rice, ragi, jowar grains, and sand × 5-7 minutes), motor control and co-ordination (ball rolls under palm, picking up the coins, thread the beads – 5-7 minutes, finger to nose exercise, finger to finger exercise, buttoning and unbuttoning exercise for 5-7 minutes, standing with both feet together, walking on the line/heel toe walking, single leg stance, backward walking, stair walking, side walking for 10 minutes), strength and endurance (by using 500 ml water bottles perform, for both shoulders, elbows, and hand flexion-extension, rolling movement, wrist movements, in sitting position heel and toe raises, picking up the objects by using toes) along with balance training (static-holding an chair, heel raises, side stepping, standing with both feet together, dynamic – walking on the line/heel toe walking, single leg stance backward walking, sit to stand × 5 minutes, task-specific training – step onto a 4 inch platform by holding the rails or walls × 10 times per leg × 2 times, progression is with holding a light object and increase the inch up to 8 inches) and functional reintegration programs (by using 500 ml water bottles – rolling movements wrist curl, wrist extension for both the hands, pinch and release by finger any pencil and pen, pen spin, coin drop and pick up, finger opposition, squeezing the soft balls with hands and pressing the same ball under the feet × 5 minutes, occupational task practice). Interventional group (Group-A) was specifically designed to target the sensory and motor impairments associated with CIPN and were designed by using Specific Adaptations to Imposed Demands (SAID) Principle. The SAID principle is based on the specificity of training concepts, the physical stress theory (PST), and Wolff’s Law. The Physical Stress Theory (PST)^10^ posits that tissues adapt based on the magnitude and type of physical stress they experience, while Wolff’s Law states that bone remodels in response to the mechanical loads placed upon it, becoming stronger with increased stress and weaker with disuse.^10^ Each exercise session occurred 4 times a week, with 1-minute to 2 minutes breaks between each phase. Patients attended an initial one-on-one training session where they were provided with an exercise catalogue featuring illustrated cards, step-by-step instructions, and adherence tracking cards. Control group participants were asked to continue the usual care (walking for 35 minutes per session, flexibility exercise with both upper limb and lower limb muscle stretching along with ankle toe movements and calf muscle squeezing × 5 minutes per session with functional activities such as household works which includes cooking, staircase climbing, washing vessels, cutting vegetables; all these sessions will be done for 4 times a week × 12 weeks). Intensity for this programme was based on the rating of perceived exertion (RPE)^11^ rating scale and its intensity was ranging 3 to 5 (moderate) as our participants was under weekly paclitaxel regimen therapy. Patients were educated on the importance of physical activity for maintaining overall health and encouraged to stay engaged in routine activities. No additional exercise was prescribed. Interventional group (group A) participants were also allowed to continue the usual care. Although basic physical activity like walking may confer general health benefits, its scope and intensity are distinct from the targeted therapeutic effects of the targeted intervention evaluated in our study. Walking performance involves multiple physiological domains – such as cardiorespiratory fitness, muscular strength, motor control, and dynamic balance – and may positively influence quality of life. Therefore, it is recommended to all patients as part of routine standard of care in oncology.

A diary was maintained to record daily exercise activities, and adherence was assessed using the Exercise Adherence Rating Scale.We have used EARS Kn, a cross-culturally adapted patient-reported outcome measures (PROM) which is a translated version of the original version by Newman-Beinart et al.^12^ This is a self-reported 16 item questionnaire, consisting of 3 sections – Section A, Section B, and Section C. Section A consists of 5 – item scale wherein where in participants qualitatively explain their adherence behavior. The second section, section “B,” consisting of 6 items, is an actual measure of exercise adherence, and is used to measure adherence behavior. Furthermore, the third section – section “C” is a 10-item scale describing the reasons for adherence/non-adherence to exercise^13^ The EARS Kn total score was 24, for which 17 is the cut off value. Higher values indicate good adherence.^13^

The exercise intervention for participants, regardless of group allocation, was designed to align with the duration of their paclitaxel therapy. Both groups continued their respective exercise regimen until their final chemotherapy session and their first follow-up visit post-chemotherapy. Exercises were performed at home, with supervision provided by caregivers. The exercise program followed a “chemotherapy-periodized”^14^ approach, with prescriptions targeted exercise to the chemotherapy cycle. This method enhances physical outcomes, improve adherence and reduces adverse events. Participants were closely monitored during each cycle of chemotherapy for nutritional status (eg, hemoglobin and albumin levels), history of falls, exercise compliance, and any adverse events related to the exercise regimen.

### Outcome Measures

The primary efficacy outcome measures included latency, CMAP amplitude, SNAP Amplitude and nerve conduction velocity based on nerve conduction studies in the ulnar nerve, common peroneal nerve (CPN), and sural nerve at 3-months post paclitaxel therapy. For the purpose of this study, a reduction in CMAP amplitude by ≥30% and SNAP amplitude by ≥25% from baseline to repeat measurements at 3 m/s in the same subject was considered as significant electrophysiological evidence of neuropathy. The highest value of the paired nerve was recorded at baseline, and for follow-up visits, the same limb and nerve were measured. During each paclitaxel therapy cycle, participants were monitored using the National Cancer Institute’s Common Terminology Criteria for Adverse Events: Neuropathy and Pain (CTCAE grading Version 5.0) – for CIPN grading.^15^

The secondary outcome measures included the balance scores based on the Fullerton Advanced Balance Scale (FAB)^16^ and the taxane-induced toxicity-related quality-of-life score based on the version-4 of the Functional Assessment of Cancer Therapy–Taxane (FACT – Taxane) tool^17^ at 3-months post-paclitaxel therapy. The FAB scale score ranges from 0 to 40, with higher scores indicating better balance. A cutoff score of 16 indicated balance issues, with scores below 9 specifically classified as “impaired balance,” suggesting a higher risk of balance-related problems or falls^16^ Similarly, for the FACT – Taxane tool, a higher score indicated a better quality of life with fewer adverse effects^17^ The European Organisation for Research and Treatment of Cancer Quality of Life Questionnaire (EORTC QLQ) CIPN20^18^ was also used to assess CIPN symptoms at follow up (after 3 months) post paclitaxel therapy. Out of a possible score of 40, higher scores indicated a worsening quality of life.

In case of cyclical fatigue variations or other chemotherapy-related toxicities, participants were asked for linear progression with adjustments to volume and intensity based on individual tolerance as a safety measure. In this study, participants did not report any adjustments to the linear progression.

### Sample Size

The sample size was estimated to be 132 (66 in each group), accounting for a 25% dropout rate. This calculation was based on an anticipated effect size of 0.39, a standard deviation of 5.02, with 80% power, and a 95% confidence level derived based on the values from first 7 patients who were enrolled in the study.

### Randomization and Blinding

The study was an open-label study and the randomization sequence was generated by an independent statistician. A block randomization with a fixed block size of 10 was used and the participants were allocated to the 2 groups in a 1:1 ratio. Allocation concealment was done using the Serially Numbered Opaque Sealed Envelope (SNOSE) technique. The study investigators implemented the process of randomization once eligibility was confirmed.

### Study Procedures

After obtaining written informed consent, potential participants were screened for eligibility and were randomized to either of the 2 groups. All the participants had undergone baseline nerve conduction studies and FAB to rule out the underlying neuropathy in both sensory and motor nerve . Participants in both the groups were requested to continue their respective exercise programs until first follow up (after 3 months) post paclitaxel therapy completion. All measures were evaluated at 3 time points: T0 – Baseline (before paclitaxel administration), T1 – after completion of paclitaxel therapy, and T2– after 3 months from T1.

### Statistical Methods

An intention to treat analysis was performed using the data of all randomized participants including those who were lost to follow-up until the time they were in the study. No imputations were done to treat missing values and the statistical significance was set at *P* < .05. Baseline characteristics were presented using descriptive statistics. Continuous data were expressed as mean (standard deviation) or median (interquartile range), while categorical data were reported as frequencies and percentages. Normality of the data was assessed using the Shapiro-Wilk test. Between group analysis for the demographic variables were done using independent *t*-test or Mann-Whitney *U* test for continuous variables based on the data distribution and chi-squared test or Fisher’s exact test for categorical variables. Within-group analysis was performed using Friedman’s test, followed by post-hoc Dunn’s tests. A simple Bonferroni’s correction was applied to the *P*-value to account for multiple comparisons.

Multivariable analyses (Interventional group with reference to the control group) for the latency, amplitude and velocity variables were conducted using Generalized Linear Models (GLM), adjusting for the baseline measurements and other demographic variables that were significantly different between the groups at baseline. For each of these variables, the adjusted analysis was performed twice: first, with the value at the last chemotherapy session as the dependent variable and baseline measurements along with significant demographic variables as predictors; and second, with the follow-up level as the dependent variable, using baseline and last chemotherapy measurements along with significant demographic variables such as the molecular phenotype, highest recorded hemoglobin and serum albumin levels as predictors. Similarly, multivariable analyses were performed for the quality-of-life variables following the same approach. Data entry was done using Microsoft Excel (Publisher: Microsoft Corporation, Redmond, Washington, USA, 2016) and all analyses were performed using Statistical Product and Service Solutions (SPSS) for Windows, Version 25.0 (Publisher: IBM Corp., USA, 2011).

## Results

**Figure 1.**
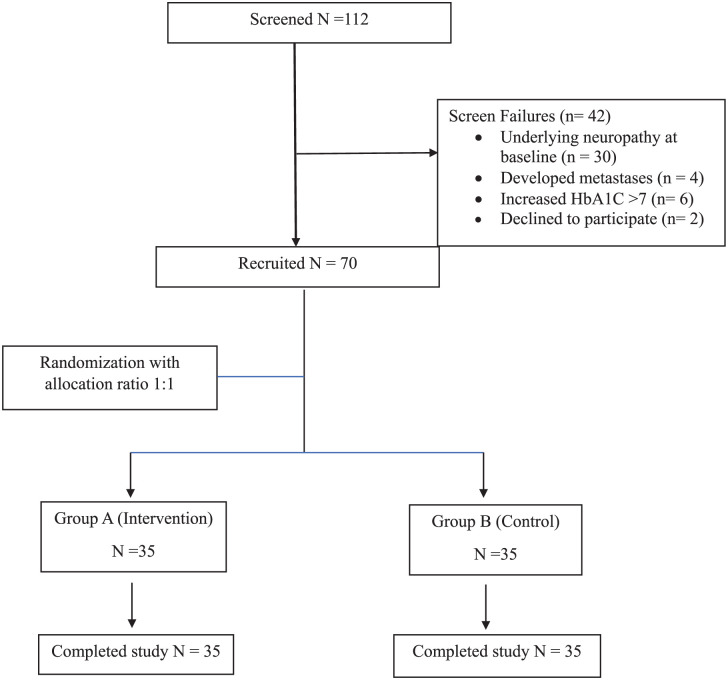
Participant flow diagram.

Out of 112 potential participants screened, 70 were successfully enrolled in the study. The primary reasons for screening failures included 30 participants presenting with underlying neuropathy based on baseline nerve conduction study recording, 6 participants with steroid-induced hyperglycemia leading to HbA1C levels above 7 during ongoing chemotherapy, 4 participants had metastatic cancer, and 2 participants declined to participate. The participant flow diagram is depicted in Figure 1. The mean (SD) age of participants was 49.46 (9.52) years in the interventional group and 49.09 (8.10) years in the control group. Luminal A was the predominant molecular phenotype (82.9%), with a significantly higher proportion in the control group than the interventional group (*P* = .034). Estrogen receptor positivity was more frequent in the control group (80%) compared to the interventional group (57.14%), approaching statistical significance (*P* = .072). The hemoglobin levels and serum albumin levels were significantly higher in the interventional group (*P* = .048 and .027, respectively). Baseline characteristics, including HER2 expression, tumor and node staging, and treatment modalities, are detailed in Table 1

**Table 1.** Baseline Demographic Characteristics.

Categorical variables								
	Levels	Overall		Group A		Group B		*P*-value
		n	%	n	%	n	%	
Molecular phenotype	Luminal A	7	10.0	2	5.71	5	14.29	.034
	Luminal B	58	82.9	28	80.00	30	85.71	
	Non luminal	5	7.1	5	14.29	0	.00	
Human epidermal growth factor receptor	HER2 neu 0	12	17.1	7	20.00	5	14.29	.535
	HER2 neu 1+	15	21.4	6	17.14	9	25.71	
	HER2 neu 2+	25	35.7	11	31.43	14	40.00	
	HER 2 neu 3+	18	25.7	11	31.43	7	20.00	
Fluorescent in situ hybridization	Negative	38	54.3	18	51.43	20	57.14	.810
	Positive	32	45.7	17	48.57	15	42.86	
Estrogen receptor	Negative	22	31.4	15	42.86	7	20.00	.072
	Positive	48	68.6	20	57.14	28	80.00	
Progesterone receptor	Negative	28	40.0	17	48.57	11	31.43	.223
	Positive	42	60.0	18	51.43	24	68.57	
Triple negative breast cancer	No	57	81.4	26	74.29	31	88.57	.219
	Yes	13	18.6	9	25.71	4	11.43	
Stage^a^	I	12	17.14%	3	8.57%	9	25.71%	.164
	II	29	41.43%	16	45.71%	13	37.14%	
	III	29	41.43%	16	45.71%	13	37.14%	
Chemotherapy	Adjuvant	26	37.14	14	40.00	12	34.29	.805
	Neo-adjuvant	44	62.86	21	60.00	23	65.71	
Menopausal status	Perimenopausal	23	32.9	13	37.14	10	28.57	.283
	Postmenopausal	27	38.6	15	42.86	12	34.29	
	Premenopausal	20	28.6	7	20.00	13	37.14	
Marital status	Married	69	98.6	35	100.00	34	97.14	1.000
	Unmarried	1	1.4	0	0.00	1	2.86	
Invasive Ductal Carcinoma, Not Otherwise Specified (IDC-NOS) grade	1	9	12.9	3	8.57	6	17.14	.634
	2	25	35.7	13	37.14	12	34.29	
	3	36	51.43	19	54.29	17	48.57	
Surgery	Lumpectomy	5	7.1	5	14.29	0	0.00	.054
	Total mastectomy	65	92.9	30	85.71	35	100.00	
Only paclitaxel	No	46	65.7	24	68.57	22	62.86	.801
	Yes	24	34.3	11	31.43	13	37.14	
Paclitaxel + trastuzumab	No	36	51.4	18	51.43	18	51.43	1.000
	Yes	34	48.6	17	48.57	17	48.57	
Paclitaxel + carboplatin	No	58	82.9	28	80.00	30	85.71	.751
	Yes	12	17.1	7	20.00	5	14.29	
Radiotherapy	No	14	20.0	4	11.43	10	28.57	.135
	Yes	56	80.0	31	88.57	25	71.43	
Continuous variables								
		Mean	SD	Mean	SD	Mean	SD	*P*-value
Age (years)		49.27	9.52	49.46	8.10	49.09	10.88	.872
Hemoglobin – Highest (g%)		12.17	1.09	12.42	0.93	11.92	1.19	.048
Hemoglobin – Lowest (g%)		10.24	1.20	10.37	1.14	10.10	1.26	.352
Albumin (mg%)		4.43	0.31	4.51	0.26	4.35	0.24	.027
Body surface area (m^2^)		1.53	0.17	1.53	0.17	1.52	0.16	.859
Paclitaxel dose (mg)		119.19	13.39	119.23	13.17	119.14	13.80	1.000

a In this cohort, breast cancer staging was classified as per TNM classification (American Joint Committee on Cancer (AJCC) staging manual, eighth edition), Neo-adjuvant chemotherapy (NACT) was based on clinical staging, and Adjuvant chemotherapy (Adj. CT) was based on pathological staging.

Within-group analysis of nerve conduction parameters and post-hoc results are summarized in Supplemental Tables 1 and 2, respectively. Percentage changes in these parameters from T0 to T1 and T1 to T2 for the overall cohort and groups are in Supplemental Table 3. From T0 to T1, there was a significant fall in the amplitudes of CPN and sural nerves in both the groups and a significant fall in their velocities was recorded only in the control group. The amplitudes of the CPN and sural nerves significantly declined in both groups, with greater reductions in the control group (−54.84% and −57.32%) compared to the intervention group (−37.27% and −26.46%). Velocity decreased moderately across all nerves, most notably in the control group. Latency generally increased, with a notable rise in sural nerve latency in the control group (+7.21%). These findings indicate greater nerve conduction impairments at T1 in the control group. From T1 to T2, amplitudes improved across all nerves, with the intervention group showing significant improvements, particularly in the sural (84.90%) and CPN (68.01%) nerves both of which were statistically significant. The control group also showed significant improvement in CPN amplitude (52.55%) but to a lesser extent. Velocity increased significantly in the intervention group for the CPN nerve (5.8%), while the control group showed a slight decline (−1.49%).

The multivariable analysis of nerve conduction studies showed significant findings for amplitude of all the 3 nerves – ulnar, common peroneal, and sural nerves. At the time of completion of paclitaxel therapy, amplitude of the ulnar nerve (estimate: 1.98, *P* = .007), amplitude of the CPN (estimate: 1.88, *P* = .003) and amplitude of sural nerve (estimate: 6.20, *P* < .001) were significantly higher in the intervention group when compared to the control group. In addition, velocity of the common peroneal nerve (estimate: 1.93, *P* = .045) and the sural nerve (estimate: 3.70, *P* = .008) were also significantly higher in the intervention group. At the follow-up 3 months after completion of paclitaxel therapy, the amplitude of the common peroneal nerve remained significantly higher in the intervention group (estimate: 3.31, *P* < .001), while the amplitude of the sural nerve was also higher in the intervention group but did not reach statistical significance. The amplitude of the ulnar nerve was similar in both groups. With regards to the velocity, all the 3 nerves had a higher velocity in the intervention group but did not reach statistical significance. There was no significant difference in latency at both time points on all the 3 nerves between the 2 groups. The details of the univariate analysis and the individual multivariable analysis models for latency, amplitude and velocity for all the 3 nerves are detailed in Supplemental Tables 4 and 5. A summary of the adjusted values of these variables alone (without the predictors) are depicted in Table 2.

**Table 2.** Multivariable Analysis of Nerve Conduction Studies’ Measures.

Variables	Dependent variable: Value at the time of completion of paclitaxel therapy^a^				Dependent variable: Value after 3 mo post completion of paclitaxel therapy^b^			
	Estimate	95% CI LL	95% CI UL	*P*-value	Estimate	95% CI LL	95% CI UL	*P*-value
Latency Ulnar	−0.01	−0.12	0.10	.892	0.07	−0.06	0.20	.286
Amplitude Ulnar	1.98	0.58	3.38	.007	−0.01	−1.25	1.24	.992
Velocity Ulnar	0.74	−3.02	4.51	.700	2.09	0.01	4.17	.054
Latency CPN	0.10	−0.11	0.31	.358	<0.001	−0.24	0.23	.980
Amplitude CPN	1.88	0.70	3.06	.003	3.31	1.79	4.83	<.001
Velocity CPN	1.93	0.08	3.78	.045	4.13	−0.20	8.46	.067
Latency Sural	−0.15	−0.32	0.01	.064	−0.13	−0.34	0.08	.226
Amplitude Sural	6.20	3.34	9.05	<.001	3.42	−1.29	8.12	.160
Velocity Sural	3.70	1.07	6.34	.008	1.47	−2.15	5.09	.429

Abbreviations: CI, confidence interval; LL, lower limit; UL, upper limit; CPN, common peroneal nerve.

a Adjusted for Molecular phenotype (Luminal A/Luminal B/non-luminal), Highest recorded hemoglobin, serum albumin, and baseline value.

b Adjusted for Molecular phenotype (Luminal A/Luminal B/non-luminal), Highest recorded hemoglobin, serum albumin, baseline value, and value at the time of completion of paclitaxel therapy.

In the multivariable analysis of quality-of-life measures, for the EORTC scale, where lower scores indicate better quality of life, sensory, and motor scores significantly decreased in the interventional group by the end of paclitaxel therapy (−5.46 and −3.82, *P* < .001), indicating improvements that were sustained at T2 (−1.99, *P* = .001 and −4.20, *P* < .001, respectively). Autonomic scores remained unchanged at both time points (*P* = .399). Overall EORTC scores also decreased significantly in the interventional group at the end of therapy (−9.29, *P* < .001) and showed continued improvements till follow up 3 months post-chemotherapy (−6.64, *P* < .001). The FAB scores improved significantly at T1 (1.31, *P* < .001) and were sustained at T2 (*P* < .001) in the interventional group. The FACT Taxane scale scores revealed mixed results. Physical well-being (PWB) and the FACT-G score (indicating general health) improved significantly at T1 (0.51, *P* < .001 and −1.28, *P* = .004, respectively) in the interventional group, while social well-being (SWB) declined significantly (−1.85, *P* < .001). Other components showed no meaningful changes at T1. At T2, PWB and FACT-G showed sustained and statistically significant better scores than the control group, while SWB showed the opposite trend. Additionally, at T2, functional well-being (FWB) and FACT-total scores were significantly better in the interventional group compared to the control group. The details of the univariate analysis and the individual multivariable analysis models for each of the quality-of-life measures and their subscales are recorded in Supplemental Tables 6 and 7. A summary of the adjusted values of these variables alone (without the predictors) are given in Table 3.

**Table 3.** Multivariable Analysis of Quality-of-Life Measures.

Quality of life scale	Variables	Dependent variable: Value at the time of completion of paclitaxel therapy^a^				Dependent variable: Value at after 3 mo post completion of paclitaxel therapy^b^			
		Estimate	95% CI LL	95% CI UL	*P*-value	Estimate	95% CI LL	95% CI UL	*P*-value
EORTC scale	Sensory	−5.46	−5.83	−5.09	<.001	−1.99	−3.15	−0.83	.001
	Motor	−3.82	−4.32	−3.33	<.001	−4.20	−4.71	−3.68	<.001
	Autonomic	<0.01	<0.01	<0.01	.399	<0.01	<0.01	<0.01	.399
	Overall	−9.29	−9.93	−8.64	<.001	−6.64	−8.03	−5.24	<.001
FAB scale		1.31	0.91	1.70	<.001	<0.01	<0.01	<0.01	<.001
FACT TAXANE scale	PWB	0.51	0.31	0.71	<.001	<0.01	<0.01	<0.01	<.001
	SWB	−1.85	−2.49	−1.21	<.001	<0.01	<0.01	<0.01	<.001
	EWB	<0.01	<0.01	<0.01	.190	<0.01	<0.01	<0.01	.190
	FWB	−0.02	−0.57	0.54	.953	<0.01	<0.01	<0.01	<.001
	TAX S	−1.08	−4.42	2.26	.530	<0.01	<0.01	<0.01	.982
	FACT TOI	−1.03	−3.93	1.88	.492	<0.01	<0.01	<0.01	.967
	FACT G	−1.28	−2.11	−0.44	.004	<0.01	<0.01	<0.01	<.001
	FACT total	−2.59	−5.51	0.34	.088	<0.01	<0.01	<0.01	<.001

Abbreviations: CI, confidence interval; LL, lower limit; UL, upper limit; EORTC, European Organisation for Research and Treatment of Cancer Quality of Life; FAB, Fullerton Advanced Balance Scale; FACT Taxane, Functional Assessment of Cancer Therapy–Taxane; PWB, physical well-being; SWB, social/family well-being; EWB, emotional well-being; FWB, functional well-being; TAX S, Taxane-specific subscale; FACT TOI, Functional Assessment of Cancer Therapy Trial Outcome Index; FACT G, Functional Assessment of Cancer Therapy – General; FACT Total, Total FACT-Taxane Score (sum of FACT G and FACT S).

a Adjusted for Molecular phenotype (Luminal A/Luminal B/non-luminal), Highest recorded hemoglobin, serum albumin, and baseline value (Baseline value not included in the EORTC assessments alone as they were exactly the same value across both the groups).

b Adjusted for Molecular phenotype (Luminal A/Luminal B/non-luminal), Highest recorded hemoglobin, serum albumin, baseline value, and value at the time of completion of paclitaxel therapy.

Adherence data demonstrated that the Group A (Interventional group) and Group B (Usual care) median adherence rate was 17 and 18 respectively out of 24, which is the maximum score, with an IQR of 1 and 0 respectively. The overall median was 18 with the IQR 1. These findings indicates that the group B had a slightly higher median exercise adherence rate compared to group A.

## Discussion

In this study, we present the results of the interim analysis of the ExPIN trial, an ongoing open-label, parallel-group RCT designed to assess the efficacy of exercise intervention on the severity of CIPN and quality of life in breast cancer patients undergoing paclitaxel treatment. The intervention was implemented as a prehabilitation method. We report that the intervention group exhibited significantly higher amplitudes values for the ulnar, CPN, and sural nerves at the end of paclitaxel therapy, with some sustained improvements at after 3-month follow-up post chemotherapy completion. However, mixed results were observed in the latency and velocity values between the 2 groups. Additionally, quality-of-life measures such as the EORTC and FAB scores showed beneficial and sustained improvements, however the FACT-taxane scale scores showed mixed results. However, these findings are preliminary and should be interpreted with caution, as the final data may provide a more comprehensive understanding

Taxanes are known to cause peripheral neuropathy by disrupting microtubule function in neurons, leading to nerve damage, inflammation, and impaired sensory and motor function. In nerve conduction studies, amplitude is often more affected than velocity or latency because taxane-induced neuropathy primarily affects the sensory and motor nerve fibers, reducing the signal strength (amplitude) rather than altering the speed (velocity) or delay (latency) of the nerve impulses.^19^ This is due to the axonal damage caused by the drug, which impairs the ability of nerve fibers to transmit signals effectively^20^ thereby explaining the significant changes in amplitude that were observed in this study compared to changes in latency or velocity. Few prior RCT’s have demonstrated the beneficial effects of exercise in mitigating neurotoxic effects of chemotherapeutic agents, especially taxanes in patients diagnosed with breast cancer^21,22^ Few other studies have also demonstrated benefit with different physical activity modalities such as resistance training and yoga.^22,23^ However, these studies are limited by their small sample size and lack of objective outcome measures such as the nerve conduction studies, both of which have been addressed in the current study.

Exercise has been shown to enhance peripheral blood circulation, which may improve nerve health and alleviate symptoms of neuropathy. Regular aerobic exercise can enhance vascular function, reduce ischemia, and promote nerve regeneration, potentially reducing the severity of chemotherapy-induced peripheral neuropathy (CIPN).^24^ Oxidative stress and inflammation are key mechanisms in CIPN, and exercise has been linked to a reduction in both by downregulating inflammatory cytokines, which could help mitigate CIPN caused by paclitaxel.^25^ Additionally, physical activity stimulates the release of endorphins and neurotrophic factors, which may modulate pain perception and improve tolerance to neuropathic symptoms.^26^ Exercise may also support neurological plasticity stimulating the brain’s adaptive capacity by increasing the production of growth factors like brain-derived neurotrophic factor (BDNF), which aids in the repair of damaged peripheral nerves.^27^

Given the beneficial effects of exercise in reducing the symptoms of CIPN, exercise could potentially serve as an adjuvant to pharmacological treatments for CIPN as an alternative or a complement by addressing the root causes of CIPN such as nerve damage and circulation issues.^28^ Drugs like duloxetine or gabapentin are commonly used for managing neuropathy symptoms, but they are not free of adverse effects.^29^ Thus, exercise interventions may offer cost-effective solutions to managing CIPN potentially reducing the need for expensive pharmacological treatments, additional management strategies to mitigate the adverse effects of these pharmacological agents, or hospital admissions related to severe CIPN symptoms. Further, exercise not only improves physical functioning but could potentially serve as a holistic management approach for the overall well-being of cancer patients, including improvements in depression and anxiety leading to better quality of life among the breast cancer patients with paclitaxel chemotherapy who participated in exercise interventions.^30^ For instance, Kleckner et al,^31^ in their pilot randomized trial of 19 cancer patients, found that 12 weeks of moderate exercise during neurotoxic chemotherapy reduced nerve-related symptoms (CIPN-20 score dropped by 7.9 at mid-treatment and 4.8 at end), improved touch sensitivity and leg strength, and altered brain connectivity patterns on MRI scans linked to how the body senses discomfort. Additionally, these exercise programs offer flexibility to personalize and tailor the physical activity considering consider the patient’s baseline physical function, chemotherapy regimen, and side effect profile.^32^ Supporting this, Sekaran et al, in their meta-analysis, reported that structured exercise interventions – including aerobic activity, strength training, and balance exercises – can enhance physical function, alleviate neuropathic symptoms, and improve quality of life. Additionally, sensorimotor training appears promising for modulating chemotherapy-induced peripheral neuropathy (CIPN) with no major safety concerns.^33^

The strengths of our study are that it has a comparatively large sample size in comparison to the already published studies and addresses an important clinical issue – CIPN which affects a significant proportion of breast cancer patients. Our study findings suggest a substantial impact on improving quality of life. Also, our study assesses efficacy using objective measures as well as patient reported outcome measures (PROMs) thereby providing a holistic evaluation of the intervention’s impact. The use of multivariable analyses to adjust for potential confounders and baseline differences enhances the robustness of the findings and helps to control for confounding variables.

Our study has some limitations. The open-label design may have introduced bias in the PROMs. However, we mitigated this potential impact by incorporating objective clinical measures, such as nerve conduction studies, which helped corroborate the findings from the PROMs. Additionally, while the study was conducted in a tertiary care hospital in coastal Karnataka, the results may not be easily generalizable to other populations or healthcare settings, particularly in rural areas or countries with differing healthcare systems. A subset of patients in our study received carboplatin in combination with paclitaxel. Given that carboplatin is also known to cause peripheral neuropathy, its contribution to the neuropathy scores observed cannot be ruled out and may represent a potential confounding factor. Due to sample size limitations, a subgroup analysis was not feasible. Nevertheless, our study was designed to reflect real-world treatment scenarios, where such drug combinations are commonly used. Similarly, the intensity of the exercise regimen was not standardized, in order to align with the variability seen in clinical practice, particularly considering the fatigue associated with cancer, paclitaxel, and other chemotherapeutic agents. Future studies with stratified randomization and larger sample sizes are warranted to better isolate the individual effects of each agent.

In summary, the current study demonstrates that an exercise intervention, when incorporated alongside paclitaxel chemotherapy, can significantly reduce the severity of CIPN in breast cancer patients. Specifically, the intervention group showed improved nerve conduction parameters, especially amplitude and better balance scores when compared to the control group. Moreover, participants in the intervention group exhibited sustained improvements in quality of life, particularly in terms of sensory and motor symptoms related to CIPN. These findings support the potential of exercise as an effective and feasible strategy to manage CIPN, enhance physical function, and improve quality of life in breast cancer survivors undergoing paclitaxel therapy. However, these findings are preliminary and require confirmation with the final data once the study is complete. More multi-centered research is needed with a wider range of neurotoxic chemotherapeutic agents and longer follow-up periods with different groups of people to confirm these results and investigate the long-term benefits of exercise interventions in this group.

## Supplemental Material

sj-docx-1-ict-10.1177_15347354251398002 – Supplemental material for Efficacy of Exercise on Severity of Paclitaxel-Induced Peripheral Neuropathy and Improving Quality of Life in Women with Non-metastatic Breast Cancer: Results of an Interim Analysis from an Ongoing Randomized Clinical Trial (ExPIN Trial) Supplemental material, sj-docx-1-ict-10.1177_15347354251398002 for Efficacy of Exercise on Severity of Paclitaxel-Induced Peripheral Neuropathy and Improving Quality of Life in Women with Non-metastatic Breast Cancer: Results of an Interim Analysis from an Ongoing Randomized Clinical Trial (ExPIN Trial) by Mangaiyarkarasi Sekaran, Ananth Pai, Arvind N. Prabhu, Vasudeva Bhat K, Chethan Channaveera, Jeffrey Pradeep Raj, Vijetha Shenoy Belle, Sharada Mailankody and Karthik S. Udupa in Integrative Cancer Therapies

## References

[1] Hershman DL Weimer LH Wang A , et al Association between patient reported outcomes and quantitative sensory tests for measuring long-term neurotoxicity in breast cancer survivors treated with adjuvant paclitaxel chemotherapy. Breast Cancer Res Treat. 2011;125(3):767-774. 21128110 10.1007/s10549-010-1278-0

[2] Bandos H Melnikow J Rivera DR , et al Long-term peripheral neuropathy in breast cancer patients treated with adjuvant chemotherapy: NRG Oncology/NSABP B-30. J Natl Cancer Inst. 2018;110(2):djx162. 10.1093/jnci/djx162 PMC5825682 28954297

[3] Sparano JA Wang M Martino S , et al Weekly paclitaxel in the adjuvant treatment of breast cancer. N Engl J Med. 2008;358(16):1663-1671 (Erratum in: N Engl J Med. 2008;359(1):106; N Engl J Med. 2009;360(16):1685). 18420499 10.1056/NEJMoa0707056 PMC2743943

[4] Loprinzi CL Reeves BN Dakhil SR , et al Natural history of paclitaxel-associated acute pain syndrome: prospective cohort study NCCTG N08C1. J Clin Oncol. 2011;29(11):1472-1478. 21383290 10.1200/JCO.2010.33.0308 PMC3082985

[5] Openshaw H Beamon K Synold TW , et al Neurophysiological study of peripheral neuropathy after high-dose paclitaxel: lack of neuroprotective effect of amifostine. Clin Cancer Res. 2004;10(2):461-467. 14760066 10.1158/1078-0432.ccr-0772-03

[6] Mauri D Kamposioras K Tsali L , et al Overall survival benefit for weekly vs. three-weekly taxanes regimens in advanced breast cancer: a meta-analysis. Cancer Treat Rev. 2010;36(1):69-74. 19945225 10.1016/j.ctrv.2009.10.006

[7] Seidman AD Berry D Cirrincione C , et al Randomized phase III trial of weekly compared with every-3-weeks paclitaxel for metastatic breast cancer, with trastuzumab for all HER-2 overexpressors and random assignment to trastuzumab or not in HER-2 nonoverexpressors: final results of cancer and Leukemia Group B protocol 9840. J Clin Oncol. 2008;26(10):1642-1649. 18375893 10.1200/JCO.2007.11.6699

[8] McCrary JM Goldstein D Sandler CX , et al Exercise-based rehabilitation for cancer survivors with chemotherapy-induced peripheral neuropathy. Support Care Cancer. 2019;27(10):3849-3857. 30756229 10.1007/s00520-019-04680-w

[9] Kneis S Wehrle A Müller J , et al It’s never too late - balance and endurance training improves functional performance, quality of life, and alleviates neuropathic symptoms in cancer survivors suffering from chemotherapy-induced peripheral neuropathy: results of a randomized controlled trial. BMC Cancer. 2019;19(1):414. 31046719 10.1186/s12885-019-5522-7 PMC6498676

[10] Mueller MJ Maluf KS. Tissue adaptation to physical stress: a proposed “physical stress theory” to guide physical therapist practice, education, and research. Phys Ther. 2002;82(4):383-403. 11922854

[11] Grummt M Hafermann L Claussen L Herrmann C Wolfarth B. Rating of perceived exertion: a large cross-sectional study defining intensity levels for individual physical activity recommendations. Sports Med Open. 2024;10(1):71. 38856875 10.1186/s40798-024-00729-1 PMC11164849

[12] Newman-Beinart NA Norton S Dowling D , et al The development and initial psychometric evaluation of a measure assessing adherence to prescribed exercise: the Exercise Adherence Rating Scale (EARS). Physiotherapy. 2017;103(2):180-185. 27913064 10.1016/j.physio.2016.11.001

[13] Pai HD Kumar KV Mithra P Samuel SR Athiyamaan MS Godfrey EL. Translation, cross-cultural adaptation, and validation of the Kannada version of the exercise adherence rating scale (EARS-Kn) among head and neck cancer (HNC) survivors in a tertiary care setup in India. Integr Cancer Ther. 2025;24:15347354251313534. 10.1177/15347354251313534 PMC11733881 39811882

[14] Kirkham AA Bland KA Zucker DS , et al “Chemotherapy-periodized” exercise to accommodate for cyclical variation in fatigue. Med Sci Sports Exerc. 2020;52(2):278-286. 31490858 10.1249/MSS.0000000000002151

[15] National Cancer Institute. Common Terminology Criteria for Adverse Events (CTCAE) version 5.0. U.S. Department of Health and Human Services; 2017. Accessed December 4, 2024. https://ctep.cancer.gov/protocoldevelopment/electronic_applications/ctc.htm

[16] Pooranawatthanakul K Siriphorn A. Accuracy of the Fullerton Advanced Balance (FAB) scale and a modified FAB model for predicting falls in older adults: a prospective study. J Bodyw Mov Ther. 2023;36:393-398. 37949590 10.1016/j.jbmt.2023.09.001

[17] Cella D Peterman A Hudgens S Webster K Socinski MA. Measuring the side effects of taxane therapy in oncology: the functional assesment of cancer therapy-Taxane (FACT-taxane). Cancer. 2003;98(4):822-831. 12910528 10.1002/cncr.11578

[18] Rattanakrong N Thipprasopchock S Siriphorn A Boonyong S. Reliability and validity of the EORTC QLQ-CIPN20 (European Organization for research and treatment of Cancer Quality of life questionnaire-chemotherapy-induced peripheral neuropathy 20-Item scale) among Thai women with breast cancer undergoing taxane-based chemotherapy. Asian Pac J Cancer Prev. 2022;23(5):1547-1553. 35633537 10.31557/APJCP.2022.23.5.1547 PMC9587879

[19] Velasco R Bruna J. Taxane-induced peripheral neurotoxicity. Toxics. 2015;3(2):152-169. doi:10.3390/toxics3020152 29056655 PMC5634686

[20] Park SB Goldstein D Krishnan AV , et al Chemotherapy-induced peripheral neurotoxicity: a critical analysis. CA Cancer J Clin. 2013;63(6):419-437. 24590861 10.3322/caac.21204

[21] Kleckner IR Kamen C Gewandter JS , et al Effects of exercise during chemotherapy on chemotherapy-induced peripheral neuropathy: a multicenter, randomized controlled trial. Support Care Cancer. 2018;26(4):1019-1028. 29243164 10.1007/s00520-017-4013-0 PMC5823751

[22] Brownson-Smith R Orange ST Cresti N Hunt K Saxton J Temesi J. Effect of exercise before and/or during taxane-containing chemotherapy treatment on chemotherapy-induced peripheral neuropathy symptoms in women with breast cancer: systematic review and meta-analysis. J Cancer Surviv. 2025;19:78-96. 37615928 10.1007/s11764-023-01450-w PMC11813970

[23] Taspinar B Aslan UB Agbuga B Taspinar F. A comparison of the effects of hatha yoga and resistance exercise on mental health and well-being in sedentary adults: a pilot study. Complement Ther Med. 2014;22(3):433-440. 24906581 10.1016/j.ctim.2014.03.007

[24] Tofthagen C Visovsky C Berry DL. Strength and balance training for adults with peripheral neuropathy and high risk of fall: current evidence and implications for future research. Oncol Nurs Forum. 2012;39(5):E416-E424. 10.1188/12.ONF.E416-E424 PMC5385995 22940521

[25] Chung KH Park SB Streckmann F , et al Mechanisms, mediators, and moderators of the effects of exercise on chemotherapy-induced peripheral neuropathy. Cancers. 2022;14(5):1224. 35267533 10.3390/cancers14051224 PMC8909585

[26] Vieira WF Real CC Martins DO Chacur M. The role of exercise on glial cell activity in neuropathic pain management. Cells. 2025;14(7):487. 40214441 10.3390/cells14070487 PMC11988158

[27] Romero Garavito A Díaz Martínez V Juárez Cortés E Negrete Díaz JV Montilla Rodríguez LM. Impact of physical exercise on the regulation of brain-derived neurotrophic factor in people with neurodegenerative diseases. Front Neurol. 2024;15:1505879. 39935805 10.3389/fneur.2024.1505879 PMC11810746

[28] Klafke N Bossert J Kröger B , et al Prevention and treatment of chemotherapy-induced peripheral neuropathy (CIPN) with non-pharmacological interventions: clinical recommendations from a systematic scoping review and an expert consensus process. Med Sci. 2023;11(1):15. 10.3390/medsci11010015 PMC9944490 36810482

[29] Tanenberg RJ Irving GA Risser RC , et al Duloxetine, pregabalin, and duloxetine plus gabapentin for diabetic peripheral neuropathic pain management in patients with inadequate pain response to gabapentin: an open-label, randomized, noninferiority comparison. Mayo Clin Proc. 2011;86(7):615-626. 21719618 10.4065/mcp.2010.0681 PMC3127557

[30] Huang Y Tan T Liu L , et al Exercise for reducing chemotherapy-induced peripheral neuropathy: a systematic review and meta-analysis of randomized controlled trials. Front Neurol. 2024;14:1252259. Erratum in: Front Neurol. 2024;15:1378461. Front Neurol. 2024;15:1460992.39148700

[31] Kleckner IR Manuweera T Lin PJ , et al Update in: Pilot trial testing the effects of exercise on chemotherapy-induced peripheral neurotoxicity (CIPN) and the interoceptive brain system. Res Sq [Preprint]. 2024:rs.3.rs-4022351. Support Care Cancer. 2024;32(10):677. 39304604 10.1007/s00520-024-08855-y PMC12203810

[32] Twomey R Martin T Temesi J Culos-Reed SN Millet GY. Tailored exercise interventions to reduce fatigue in cancer survivors: study protocol of a randomized controlled trial. BMC Cancer. 2018;18:757. 30041626 10.1186/s12885-018-4668-z PMC6057053

[33] Sekaran M Udupa KS Alok Y Pai A Bhat KV. Effects of exercise on paclitaxel-induced peripheral neuropathy in patients with cancer: a systematic review and meta-analysis. Cancer Res Stat Treat. 2025;8(1):48-65.

