# Supplementary material for: Efficacy of Exercise on Severity of Paclitaxel-Induced Peripheral Neuropathy and Improving Quality of Life in Women with Non-metastatic Breast Cancer: Results of an Interim Analysis from an Ongoing Randomized Clinical Trial (ExPIN Trial)
Source: Integr Cancer Ther. 2025 Dec 18;24:15347354251398002. doi: 10.1177/15347354251398002 (PMC12717369; doi:10.1177/15347354251398002)
Supplement: sj-docx-1-ict-10.1177_15347354251398002 – Supplemental material for Efficacy of Exercise on Severity of Paclitaxel-Induced Peripheral Neuropathy and Improving Quality of Life in Women with Non-metastatic Breast Cancer: Results of an Interim Analysis from an Ongoing Randomized Clinical Trial (ExPIN T [file sj-docx-1-ict-10.1177_15347354251398002.docx]

**Supplementary Table – 1: Within Interventional (Group A) and Control groups (Group B) Analysis (T0 – Baseline, T1- Last chemotherapy, T2 – Follow up)**

| **Variables** | **Mean (SD)** | | | ***P*-value** | **Median (25^th^, 75^th^)** | | |
| --- | --- | --- | --- | --- | --- | --- | --- |
|  | **T0** | **T1** | **T2** |  | **T0** | **T1** | **T2** |
| **Group - A** | | | | | | | |
| Latency Ulnar | 2.36 (0.23) | 2.39 (0.22) | 2.49 (0.32) | 0.031 | 2.30 (2.20, 2.40) | 2.40 (2.20, 2.50) | 2.50 (2.30, 2.60) |
| Amplitude Ulnar | 15.20 (2.84) | 11.77 (2.90) | 14.33 (2.87) | 0.124 | 15.40 (13.55, 16.95) | 11.20 (9.70, 12.70) | 14.90 (12.60, 16.15) |
| Velocity Ulnar | 57.57 (3.86) | 55.25 (3.77) | 56.96 (3.93) | 0.806 | 57.90 (55.20, 59.70) | 54.60 (52.50, 57.50) | 57.50 (54.05, 59.05) |
| Latency CPN | 3.52 (0.35) | 3.66 (0.46) | 3.62 (0.59) | 0.090 | 3.40 (3.30, 3.80) | 3.50 (3.30, 3.80) | 3.60 (3.35, 3.80) |
| Amplitude CPN | 10.42 (5.04) | 6.42 (4.41) | 10.21 (4.43) | <0.001 | 9.50 (7.30, 12.00) | 5.60 (4.30, 6.95) | 10.80 (6.60, 11.70) |
| Velocity CPN | 48.02 (3.00) | 46.81 (3.85) | 49.00 (8.40) | 0.047 | 48.00 (46.05, 49.80) | 46.30 (44.40, 49.10) | 50.00 (46.55, 52.20) |
| Latency Sural | 2.31 (0.21) | 2.33 (0.27) | 2.38 (0.39) | 0.872 | 2.30 (2.20, 2.40) | 2.30 (2.10, 2.40) | 2.30 (2.10, 2.50) |
| Amplitude Sural | 18.45 (8.41) | 12.63 (7.21) | 16.45 (7.44) | <0.001 | 18.40 (12.00, 22.75) | 12.00 (9.20, 14.05) | 16.10 (10.90, 20.30) |
| Velocity Sural | 55.18 (2.30) | 53.71 (3.95) | 53.75 (7.50) | 0.666 | 55.60 (53.70, 56.90) | 53.70 (52.20, 56.10) | 55.10 (53.30, 57.00) |
| **Group – B** | | | | | | | |
| Latency Ulnar | 2.36 (0.24) | 2.35 (0.26) | 2.39 (0.27) | 0.390 | 2.40 (2.15, 2.5) | 2.40 (2.10, 2.55) | 2.30 (2.20, 2.50) |
| Amplitude Ulnar | 14.85 (3.40) | 9.61 (3.15) | 12.54 (3.24) | 0.072 | 14.90 (12.85, 16.80) | 9.10 (7.90, 11.60) | 11.70 (9.90, 14.35) |
| Velocity Ulnar | 57.41 (4.22) | 53.24 (9.98) | 54.49 (4.37) | 0.016 | 57.50 (54.10, 59.50) | 55.10 (51.90, 57.70) | 55.70 (50.90, 57.5) |
| Latency CPN | 3.45 (0.40) | 3.43 (0.40) | 3.43 (0.45) | 0.738 | 3.50 (3.20, 3.75) | 3.40 (3.20, 3.65) | 3.40 (3.25, 3.70) |
| Amplitude CPN | 9.42 (3.04) | 4.02 (1.56) | 5.93 (2.64) | <0.001 | 9.30 (6.85, 11.50) | 3.40 (3.20, 4.80) | 5.70 (4.00, 6.70) |
| Velocity CPN | 47.91 (3.44) | 45.56 (3.31) | 44.77 (7.34) | 0.015 | 48.30 (45.35, 49.60) | 46.20 (42.95, 47.20) | 45.60 (42.60, 47.75) |
| Latency Sural | 2.22 (0.21) | 2.37 (0.36) | 2.46 (0.44) | 0.070 | 2.20 (2.10, 2.35) | 2.30 (2.10, 2.55) | 2.30 (2.10, 2.70) |
| Amplitude Sural | 21.35 (11.87) | 7.95 (4.85) | 11.55 (8.84) | <0.001 | 18.40 (15.25, 24.10) | 5.10 (4.50, 12.90) | 6.50 (5.25, 14.90) |
| Velocity Sural | 55.13 (2.99) | 50.94 (5.91) | 51.09 (5.76) | 0.001 | 54.50 (53.40, 56.80) | 51.60 (45.90, 55.80) | 52.10 (45.75, 55.05) |

CPN – Common Peroneal Nerve

**Supplementary Table – 2: Within Interventional (Group A) and Control groups (Group B) Analysis – *Post Hoc* (T0 – Baseline, T1- Last chemotherapy, T2 – Follow up)**

| **Variable** | **Comparison** | **Difference value** | **P-value** |
| --- | --- | --- | --- |
| **Group – A** | | | |
| Latency Ulnar | T0 – T1 | -0.93 | 0.531 |
|  | T0 – T2 | -2.23 | 0.038 |
|  | T1 – T2 | -1.31 | 0.287 |
| Amplitude CPN | T0 – T1 | 4.86 | <0.001 |
|  | T0 – T2 | 0.21 | 1.000 |
|  | T1 – T2 | -4.65 | <0.001 |
| Velocity CPN | T0 – T1 | 1.41 | 0.239 |
|  | T0 – T2 | -1.59 | 0.168 |
|  | T1 – T2 | -3.00 | 0.004 |
| Amplitude Sural | T0 – T1 | 3.06 | 0.003 |
|  | T0 – T2 | 0.87 | 0.577 |
|  | T1 – T2 | -2.19 | 0.042 |
| **Group – B** | | | |
| Velocity Ulnar | T0 – T1 | 2.74 | 0.009 |
|  | T0 – T2 | 2.64 | 0.012 |
|  | T1 – T2 | -0.10 | 1.000 |
| Amplitude CPN | T0 – T1 | 7.04 | <0.001 |
|  | T0 – T2 | 4.05 | <0.001 |
|  | T1 – T2 | -2.99 | 0.004 |
| Velocity CPN | T0 – T1 | 2.70 | 0.010 |
|  | T0 – T2 | 2.68 | 0.011 |
|  | T1 – T2 | -0.03 | 1.000 |
| Amplitude Sural | T0 – T1 | 6.08 | <0.001 |
|  | T0 – T2 | 4.07 | <0.001 |
|  | T1 – T2 | -2.01 | 0.066 |
| Velocity Sural | T0 – T1 | 3.18 | 0.002 |
|  | T0 – T2 | 3.27 | 0.002 |
|  | T1 – T2 | 0.09 | 1.000 |

CPN – Common Peroneal Nerve

**Supplementary Table – 3: Mean percentage change of Nerve Conduction study measures with time (T0 – Baseline, T1- Last chemotherapy, T2 – Follow up)**

| **Variables** | **T1 – T0 average change in %** | | | **T2 – T1 average change in %** | | |
| --- | --- | --- | --- | --- | --- | --- |
|  | **Overall** | **Group A** | **Group B** | **Overall** | **Group A** | **Group B** |
| Latency Ulnar | 0.90% | 1.83% | -0.03% | 3.41% | 4.67% | 2.15% |
| Amplitude Ulnar | -27.85% | -21.44% | -34.27% | 32.02% | 25.21% | 38.82% |
| Velocity Ulnar | -5.48% | -3.80% | -7.16% | 15.33% | 3.39% | 27.27% |
| Latency CPN | 2.49% | 4.34% | 0.65% | -0.05% | -0.76% | 0.65% |
| Amplitude CPN | -46.05% | -37.27% | -54.84% | 68.01% | 83.48% | 52.55% |
| Velocity CPN | -3.41% | -2.15% | -4.68% | 1.89% | 5.28% | -1.49% |
| Latency Sural | 4.54% | 1.88% | 7.21% | 3.72% | 2.85% | 4.60% |
| Amplitude Sural | -41.89% | -26.46% | -57.32% | 84.90% | 89.00% | 80.79% |
| Velocity Sural | -5.02% | -2.53% | -7.50% | 0.74% | 0.55% | 0.92% |

**Supplementary Table – 4: Between Group unadjusted analysis of Nerve Conduction Study measures (Group A – Interventional group, Group B – Control group and Overall – Combination of Group A and Group B) (T0 – Baseline, T1- Last chemotherapy, T2 – Follow up)**

| **Variables** | **Time period** | **Mean (SD)** | | | **P-value** | **Median (25^th^,75^th^)** | | |
| --- | --- | --- | --- | --- | --- | --- | --- | --- |
|  |  | **Overall** | **Group A** | **Group B** |  | **Overall** | **Group A** | **Group B** |
| Latency Ulnar | T0 | 2.359(0.229) | 2.357(0.227) | 2.360(0.235) | 0.844 | 2.300(2.200, 2.475) | 2.300(2.200, 2.400) | 2.400(2.150, 2.500) |
|  | T1 | 2.369(0.237) | 2.389(0.215) | 2.349(0.258) | 0.448 | 2.400(2.200, 2.500) | 2.400(2.200, 2.500) | 2.400(2.100, 2.550) |
|  | T2 | 2.440(0.302) | 2.494(0.324) | 2.386(0.272) | 0.083 | 2.400(2.300, 2.575) | 2.500(2.300, 2.600) | 2.300(2.200, 2.500) |
| Amplitude Ulnar | T0 | 15.023(3.116) | 15.200(2.840) | 14.846(3.403) | 0.638 | 15.150(13.050, 17.050) | 15.400(13.550, 16.950) | 14.900(12.850, 16.800) |
|  | T1 | 10.690(3.196) | 11.774(2.896) | 9.606(3.149) | 0.004 | 10.600(8.800, 12.300) | 11.200(9.700, 12.700) | 9.100(7.900, 11.600) |
|  | T2 | 13.439(3.166) | 14.334(2.868) | 12.543(3.236) | 0.017 | 13.500(10.650, 15.600) | 14.900(12.600, 16.150) | 11.700(9.900, 14.350) |
| Velocity Ulnar | T0 | 57.487(4.014) | 57.569(3.856) | 57.406(4.220) | 0.867 | 57.700(54.625, 59.700) | 57.900(55.200, 59.700) | 57.500(54.100, 59.500) |
|  | T1 | 54.244(7.559) | 55.249(3.769) | 53.24(9.984) | 0.420 | 54.700(52.400, 57.500) | 54.600(52.500, 57.500) | 55.100(51.900, 57.700) |
|  | T2 | 55.727(4.308) | 56.963(3.926) | 54.491(4.371) | 0.015 | 55.700(52.700, 58.450) | 57.500(54.050, 59.050) | 55.700(50.900, 57.500) |
| Latency CPN | T0 | 3.487(0.377) | 3.520(0.351) | 3.454(0.403) | 0.470 | 3.450(3.300, 3.800) | 3.400(3.300, 3.800) | 3.500(3.200, 3.750) |
|  | T1 | 3.547(0.446) | 3.663(0.462) | 3.431(0.403) | 0.109 | 3.400(3.300, 3.800) | 3.500(3.300, 3.800) | 3.400(3.200, 3.650) |
|  | T2 | 3.524(0.532) | 3.623(0.594) | 3.426(0.450) | 0.122 | 3.500(3.300, 3.800) | 3.600(3.350, 3.800) | 3.400(3.250, 3.700) |
| Amplitude CPN | T0 | 9.921(4.159) | 10.423(5.035) | 9.420(3.040) | 0.565 | 9.350(6.975, 11.775) | 9.500(7.300, 12.000) | 9.300(6.850, 11.500) |
|  | T1 | 5.220(3.498) | 6.417(4.409) | 4.023(1.563) | <0.001 | 4.400(3.325, 6.175) | 5.600(4.300, 6.950) | 3.400(3.200, 4.800) |
|  | T2 | 8.070(4.213) | 10.214(4.425) | 5.926(2.642) | <0.001 | 6.600(5.225, 11.100) | 10.800(6.600, 11.700) | 5.700(4.000, 6.700) |
| Velocity CPN | T0 | 47.966(3.206) | 48.02(3.001) | 47.911(3.442) | 0.889 | 48.100(45.900, 49.600) | 48.000(46.050, 49.800) | 48.300(45.350, 49.600) |
|  | T1 | 46.184(3.617) | 46.809(3.849) | 45.560(3.307) | 0.375 | 46.250(44.325, 48.275) | 46.300(44.400, 49.100) | 46.200(42.950, 47.200) |
|  | T2 | 46.884(8.113) | 49.003(8.398) | 44.766(7.335) | <0.001 | 47.350(44.450, 51.925) | 50.000(46.550, 52.200) | 45.600(42.600, 47.750) |
| Latency Sural | T0 | 2.264(0.214) | 2.306(0.213) | 2.223(0.210) | 0.085 | 2.200(2.100, 2.400) | 2.300(2.200, 2.400) | 2.200(2.100, 2.350) |
|  | T1 | 2.354(0.312) | 2.334(0.266) | 2.374(0.355) | 0.948 | 2.300(2.100, 2.400) | 2.300(2.100, 2.400) | 2.300(2.100, 2.550) |
|  | T2 | 2.423(0.412) | 2.383(0.388) | 2.463(0.436) | 0.640 | 2.300(2.100, 2.575) | 2.300(2.100, 2.500) | 2.300(2.100, 2.700) |
| Amplitude Sural | T0 | 19.901(10.313) | 18.449(8.405) | 21.354(11.869) | 0.459 | 18.400(12.925, 23.675) | 18.400(12.000, 22.750) | 18.400(15.250, 24.100) |
|  | T1 | 10.289(6.539) | 12.629(7.212) | 7.949(4.846) | 0.004 | 9.600(4.800, 13.100) | 12.000(9.200, 14.050) | 5.100(4.500, 12.900) |
|  | T2 | 13.996(8.470) | 16.446(7.435) | 11.546(8.829) | 0.002 | 13.900(5.700, 17.750) | 16.100(10.900, 20.300) | 6.500(5.250, 14.900) |
| Velocity Sural | T0 | 55.156(2.648) | 55.183(2.297) | 55.129(2.993) | 0.932 | 55.000(53.600, 56.925) | 55.600(53.700, 56.900) | 54.500(53.400, 56.800) |
|  | T1 | 52.321(5.178) | 53.706(3.948) | 50.937(5.905) | 0.038 | 53.400(50.000, 56.100) | 53.700(52.200, 56.100) | 51.600(45.900, 55.800) |
|  | T2 | 52.420(6.769) | 53.754(7.496) | 51.086(5.755) | 0.003 | 53.900(50.575, 56.600) | 55.100(53.300, 57.000) | 52.100(45.750, 55.050) |

**Supplementary Table – 5: Between Interventional Group (Group A) and Control Group (Group B) unadjusted analysis of Quality-of-Life measures (T0 – Baseline, T1- Last chemotherapy, T2 – Follow up)**

| **Variables** | **Time period** | **Mean (SD)** | | | **P-value** | **Median (25^th^,75^th^)** | | |
| --- | --- | --- | --- | --- | --- | --- | --- | --- |
|  |  | **Overall** | **Group A** | **Group B** |  | **Overall** | **Group A** | **Group B** |
| **European Organization for Research and Treatment of Cancer Quality of Life (EORTC)** | | | | | | | | |
| Sensory | T0 | 9.000(0.000) | 9.000(0.000) | 9.000(0.000) | NA | 9.000(9.000, 9.000) | 9.000(9.000, 9.000) | 9.000(9.000, 9.000) |
|  | T1 | 16.671(2.842) | 13.943(0.338) | 19.4(0.976) | <0.001 | 16.000(14.000, 20.000) | 14.000(14.000, 14.000) | 20.000(19.000, 20.000) |
|  | T2 | 14.614(3.141) | 11.600(0.695) | 17.629(0.910) | <0.001 | 14.500(11.250, 18.000) | 11.000(11.000, 12.000) | 18.000(17.000, 18.000) |
| Motor | T0 | 8.000(0.000) | 8.000(0.000) | 8.000(0.000) | NA | 8.000(8.000, 8.000) | 8.000 (8.000, 8.000) | 8.000(8.000, 8.000) |
|  | T1 | 11.143(2.169) | 9.200(0.719) | 13.086(1.121) | <0.001 | 10.000(9.000, 13.000) | 9.000(9.000, 10.000) | 13.000(13.000, 14.000) |
|  | T2 | 11.229(3.328) | 8.000(0.000) | 14.457(1.010) | <0.001 | 9.500(8.000, 15.000) | 8.000(8.000, 8.000) | 15.000(14.000, 15.000) |
| Autonomic | T0 | 2.000(0.000) | 2.000(0.000) | 2.000(0.000) | NA | 2.000(2.000, 2.000) | 2.000(2.000, 2.000) | 2.000(2.000, 2.000) |
|  | T1 | 2.000(0.000) | 2.000(0.000) | 2.000(0.000) | NA | 2.000(2.000, 2.000) | 2.000(2.000, 2.000) | 2.000(2.000, 2.000) |
|  | T2 | 2.000(0.000) | 2.000(0.000) | 2.000(0.000) | NA | 2.000(2.000, 2.000) | 2.000(2.000, 2.000) | 2.000(2.000, 2.000) |
| Overall | T0 | 19.000(0.000) | 19.000(0.000) | 19.000(0.000) | NA | 19.000(19.000, 19.000) | 19.000(19.000, 19.000) | 19.000(19.000, 19.000) |
|  | T1 | 29.814(4.870) | 25.143(0.810) | 34.486(1.597) | <0.001 | 28.500(25.000, 35.000) | 25.000(24.500, 26.000) | 35.000(34.000, 36.000) |
|  | T2 | 27.843(6.381) | 21.600(0.695) | 34.086(1.380) | <0.001 | 26.500(21.250, 34.000) | 21.000(21.000, 22.000) | 34.000(33.000, 35.000) |
| **Fullerton Advanced Balance Scale** | | | | | | | | |
| FAB | T0 | 10.200(2.574) | 10.457(2.267) | 9.943(2.859) | 0.349 | 9.000(8.000, 12.000) | 10.000(8.000, 13.000) | 9.000(8.000, 12.000) |
|  | T1 | 7.043(2.116) | 7.857(1.167) | 6.229(2.522) | 0.002 | 7.000(5.000, 8.000) | 7.000(7.000, 8.500) | 5.000(4.000, 8.000) |
|  | T2 | 10.200(2.574) | 10.457(2.267) | 9.943(2.859) | 0.349 | 9.000(8.000, 12.000) | 10.000(8.000, 13.000) | 9.000(8.000, 12.000) |
| **Functional Assessment of Cancer Therapy–Taxane** | | | | | | | | |
| PWB | T0 | 24.486(1.271) | 24.000(1.663) | 24.971(0.169) | <0.001 | 25.000(24.250, 25.000) | 25.000(24.000, 25.000) | 25.000(25.000, 25.000) |
|  | T1 | 24.300(0.462) | 24.600(0.497) | 24.000(0.000) | <0.001 | 24.000(24.000, 25.000) | 25.000(24.000, 25.000) | 24.000(24.000, 24.000) |
|  | T2 | 24.300(0.462) | 24.600(0.497) | 24.000(0.000) | <0.001 | 24.000(24.000, 25.000) | 25.000(24.000, 25.000) | 24.000(24.000, 24.000) |
| SWB | T0 | 18.933(1.364) | 18.666(1.47) | 19.200(1.211) | 0.155 | 18.670(17.500, 19.830) | 18.670(17.500, 19.830) | 18.670(18.670, 19.830) |
|  | T1 | 18.382(1.569) | 17.399(1.635) | 19.366(0.577) | <0.001 | 18.670(16.330, 19.830) | 16.330(16.330, 18.670) | 19.830(18.670, 19.830) |
|  | T2 | 18.382(1.569) | 17.399(1.635) | 19.366(0.577) | <0.001 | 18.670(16.330, 19.830) | 16.330(16.330, 18.670) | 19.830(18.670, 19.830) |
| EWB | T0 | 11.557(1.002) | 11.829(1.043) | 11.286(0.893) | 0.029 | 12.000(11.000, 12.000) | 12.000(11.000, 13.000) | 11.000(11.000, 12.000) |
|  | T1 | 20.000(0.000) | 20.000(0.000) | 20.000(0.000) | NA | 20.000(20.000, 20.000) | 20.000(20.000, 20.000) | 20.000(20.000, 20.000) |
|  | T2 | 20.000(0.000) | 20.000(0.000) | 20.000(0.000) | NA | 20.000(20.000, 20.000) | 20.000(20.000, 20.000) | 20.000(20.000, 20.000) |
| FWB | T0 | 17.100(3.556) | 16.600(4.251) | 17.600(2.659) | 0.414 | 16.000(15.000, 21.000) | 16.000(14.500, 21.000) | 16.000(15.000, 20.500) |
|  | T1 | 26.814(1.094) | 26.657(1.162) | 26.971(1.014) | 0.225 | 26.500(26.000, 28.000) | 27.000(26.000, 28.000) | 26.000(26.000, 28.000) |
|  | T2 | 26.814(1.094) | 26.657(1.162) | 26.971(1.014) | 0.225 | 26.500(26.000, 28.000) | 27.000(26.000, 28.000) | 26.000(26.000, 28.000) |
| TAX-S | T0 | 61.643(0.660) | 61.743(0.780) | 61.543(0.505) | 0.391 | 62.000(61.000, 62.000) | 62.000(61.000, 62.000) | 62.000(61.000, 62.000) |
|  | T1 | 47.943(6.340) | 48.086(6.577) | 47.800(6.187) | 0.754 | 46.500(41.000, 55.000) | 46.000(41.000, 56.000) | 48.000(41.000, 54.500) |
|  | T2 | 47.943(6.340) | 48.086(6.577) | 47.800(6.187) | 0.754 | 46.500(41.000, 55.000) | 46.000(41.000, 56.000) | 48.000(41.000, 54.500) |
| FACT TOI | T0 | 103.229(3.535) | 102.343(4.379) | 104.114(2.139) | 0.107 | 103.000(102.000, 106.000) | 102.000(99.500, 106.000) | 103.000(102.000, 106.000) |
|  | T1 | 99.057(5.530) | 99.343(5.836) | 98.771(5.275) | 0.546 | 98.000(93.000, 105.000) | 98.000(93.500, 106.500) | 98.000(93.000, 104.500) |
|  | T2 | 99.057(5.530) | 99.343(5.836) | 98.771(5.275) | 0.546 | 98.000(93.000, 105.000) | 98.000(93.500, 106.500) | 98.000(93.000, 104.500) |
| FACT-G | T0 | 72.076(4.529) | 71.095(5.342) | 73.057(3.332) | 0.115 | 72.830(68.670, 75.670) | 70.670(67.420, 75.415) | 73.670(70.165, 75.915) |
|  | T1 | 89.497(1.759) | 88.656(1.831) | 90.337(1.213) | <0.001 | 89.750(88.372, 90.670) | 88.330(87.330, 89.670) | 89.830(89.830, 91.830) |
|  | T2 | 89.497(1.759) | 88.656(1.831) | 90.337(1.213) | <0.001 | 89.750(88.372, 90.670) | 88.330(87.330, 89.670) | 89.830(89.830, 91.830) |
| FACT – total | T0 | 133.719(3.957) | 132.837(4.657) | 134.600(2.912) | 0.105 | 134.500(130.752, 136.790) | 132.670(129.750, 136.415) | 134.830(132.165, 137.000) |
|  | T1 | 137.440(5.602) | 136.742(5.883) | 138.137(5.299) | 0.274 | 137.330(132.830, 143.165) | 135.830(132.330, 142.750) | 137.830(132.830, 143.170) |
|  | T2 | 137.440(5.602) | 136.742(5.883) | 138.137(5.299) | 0.274 | 137.330(132.830, 143.165) | 135.830(132.330, 142.750) | 137.830(132.830, 143.170) |

SD – Standard Deviation; EORTC - European Organization for Research and Treatment of Cancer Quality of Life; FAB - Fullerton Advanced Balance Scale; FACT Taxane - Functional Assessment of Cancer Therapy–Taxane; PWB – Physical Well-Being; SWB - Social/Family Well-Being; EWB – Emotional Well-Being; FWB – Functional Well-Being; TAX S - Taxane-Specific Subscale; FACT TOI – Functional Assessment of Cancer Therapy Trial Outcome Index; FACT G - Functional Assessment of Cancer Therapy – General; FACT Total: Total FACT-Taxane Score (sum of FACT G and FACT S).

**Supplementary Table – 6:** **Multivariable analysis of Nerve Conduction Study measures (Group A-Interventional group and Group B-Control group).**

| **Variables** | **Levels** | **Dependent Variable: Value at the time of completion of paclitaxel therapy^*^** | | | | **Dependent Variable: Value at 3 times post completion of paclitaxel therapy^#^** | | | |
| --- | --- | --- | --- | --- | --- | --- | --- | --- | --- |
|  |  | **Estimate** | **95% CI LL** | **95% CI UL** | **P-value** | **Estimate** | **95% CI LL** | **95% CI UL** | **P-value** |
| **Ulnar Latency** | | | | | | | | | |
| Treatment | Group A | -0.01 | -0.12 | 0.10 | 0.892 | 0.07 | -0.06 | 0.20 | 0.286 |
|  | Group B | Reference | | | | Reference | | | |
| Value at Baseline | -- | 0.48 | 0.26 | 0.70 | <0.001 | 0.47 | 0.18 | 0.75 | 0.002 |
| Value at completion of Paclitaxel | -- | Not included in the analysis | | | | 0.43 | 0.15 | 0.71 | 0.004 |
| Luminal status | Luminal A | Reference | | | | Reference | | | |
|  | Luminal B | 0.09 | -0.09 | 0.26 | 0.332 | -0.01 | -0.21 | 0.18 | 0.888 |
|  | Non-Luminal | 0.13 | -0.13 | 0.39 | 0.326 | 0.13 | -0.17 | 0.43 | 0.397 |
| Highest Haemoglobin | -- | 0.05 | 0.00 | 0.10 | 0.058 | -0.01 | -0.07 | 0.04 | 0.625 |
| Albumin | -- | 0.07 | -0.10 | 0.24 | 0.416 | 0.06 | -0.13 | 0.26 | 0.516 |
| **Ulnar Amplitude** | | | | | | | | | |
| Treatment | Group A | 1.98 | 0.58 | 3.38 | 0.007 | -0.01 | -1.25 | 1.24 | 0.992 |
|  | Group B | Reference | | | | Reference | | | |
| Value at Baseline | -- | 0.49 | 0.28 | 0.70 | <0.001 | 0.17 | -0.03 | 0.38 | 0.102 |
| Value at completion of Paclitaxel | -- | Not included in the analysis | | | | 0.58 | 0.37 | 0.79 | <0.001 |
| Luminal status | Luminal A | Reference | | | | Reference | | | |
|  | Luminal B | 0.98 | -1.17 | 3.13 | 0.373 | -0.43 | -2.26 | 1.39 | 0.642 |
|  | Non-Luminal | -0.21 | -3.46 | 3.03 | 0.897 | 1.55 | -1.19 | 4.29 | 0.271 |
| Highest Haemoglobin | -- | 0.17 | -0.44 | 0.78 | 0.593 | 0.24 | -0.27 | 0.76 | 0.360 |
| Albumin | -- | 0.10 | -2.05 | 2.26 | 0.925 | 0.71 | -1.10 | 2.53 | 0.444 |
| **Ulnar Velocity** | | | | | | | | | |
| Treatment | Group A | 0.74 | -3.02 | 4.51 | 0.700 | 2.09 | 0.01 | 4.17 | 0.054 |
|  | Group B | Reference | | | | Reference | | | |
| Value at Baseline | -- | 0.62 | 0.19 | 1.05 | 0.007 | 0.41 | 0.16 | 0.66 | 0.002 |
| Value at completion of Paclitaxel | -- | Not included in the analysis | | | | 0.02 | -0.12 | 0.15 | 0.812 |
| Luminal status | Luminal A | Reference | | | | Reference | | | |
|  | Luminal B | 2.30 | -3.52 | 8.11 | 0.442 | 0.16 | -3.07 | 3.38 | 0.925 |
|  | Non-Luminal | 2.41 | -6.43 | 11.26 | 0.595 | 0.86 | -4.03 | 5.76 | 0.730 |
| Highest Haemoglobin | -- | 1.62 | -0.01 | 3.25 | 0.056 | 0.29 | -0.64 | 1.22 | 0.544 |
| Albumin | -- | 0.86 | -4.84 | 6.55 | 0.768 | 0.15 | -3.00 | 3.29 | 0.928 |
| **Common Peroneal Nerve Latency** | | | | | | | | | |
| Treatment | Group A | 0.10 | -0.11 | 0.31 | 0.358 | -0.01 | -0.24 | 0.23 | 0.980 |
|  | Group B | Reference | | | | Reference | | | |
| Value at Baseline | -- | 0.45 | 0.18 | 0.72 | 0.002 | 0.16 | -0.16 | 0.48 | 0.321 |
| Value at completion of Paclitaxel | -- | Not included in the analysis | | | | 0.60 | 0.33 | 0.87 | <0.001 |
| Luminal status | Luminal A | Reference | | | | Reference | | | |
|  | Luminal B | 0.08 | -0.25 | 0.41 | 0.634 | 0.10 | -0.27 | 0.46 | 0.607 |
|  | Non-Luminal | 0.12 | -0.38 | 0.62 | 0.639 | 0.44 | -0.12 | 0.99 | 0.127 |
| Highest Haemoglobin | -- | 0.06 | -0.04 | 0.15 | 0.242 | 0.01 | -0.10 | 0.11 | 0.939 |
| Albumin | -- | 0.38 | 0.05 | 0.70 | 0.029 | -0.05 | -0.43 | 0.33 | 0.788 |
| **Common Peroneal Nerve Amplitude** | | | | | | | | | |
| Treatment | Group A | 1.88 | 0.70 | 3.06 | 0.003 | 3.31 | 1.79 | 4.83 | <0.001 |
|  | Group B | Reference | | | | Reference | | | |
| Value at Baseline | -- | 0.61 | 0.48 | 0.74 | <0.001 | 0.37 | 0.13 | 0.61 | 0.003 |
| Value at completion of Paclitaxel | -- | Not included in the analysis | | | | 0.38 | 0.08 | 0.67 | 0.014 |
| Luminal status | Luminal A | Reference | | | | Reference | | | |
|  | Luminal B | 0.78 | -1.04 | 2.59 | 0.405 | -0.75 | -2.93 | 1.43 | 0.501 |
|  | Non-Luminal | 1.55 | -1.19 | 4.30 | 0.271 | -0.69 | -4.00 | 2.62 | 0.685 |
| Highest Haemoglobin | -- | -0.24 | -0.75 | 0.27 | 0.367 | -0.11 | -0.72 | 0.51 | 0.738 |
| Albumin | -- | -0.95 | -2.75 | 0.84 | 0.303 | -1.18 | -3.35 | 0.98 | 0.289 |
| **Common Peroneal Nerve Velocity** | | | | | | | | | |
| Treatment | Group A | 1.93 | 0.08 | 3.78 | 0.045 | 4.13 | -0.20 | 8.46 | 0.067 |
|  | Group B | Reference | | | | Reference | | | |
| Value at Baseline | -- | 0.32 | 0.05 | 0.59 | 0.022 | -0.07 | -0.70 | 0.56 | 0.830 |
| Value at completion of Paclitaxel | -- | Not included in the analysis | | | | 0.37 | -0.19 | 0.93 | 0.202 |
| Luminal status | Luminal A | Reference | | | | Reference | | | |
|  | Luminal B | -1.69 | -4.53 | 1.15 | 0.249 | 4.55 | -1.96 | 11.06 | 0.175 |
|  | Non-Luminal | -2.17 | -6.48 | 2.13 | 0.326 | 3.18 | -6.66 | 13.01 | 0.529 |
| Highest Haemoglobin | -- | -0.46 | -1.27 | 0.35 | 0.271 | -0.40 | -2.25 | 1.45 | 0.671 |
| Albumin | -- | -1.69 | -4.52 | 1.14 | 0.247 | -2.08 | -8.57 | 4.41 | 0.533 |
| **Sural Latency** | | | | | | | | | |
| Treatment | Group A | -0.15 | -0.32 | 0.01 | 0.064 | -0.13 | -0.34 | 0.08 | 0.226 |
|  | Group B | Reference | | | | Reference | | | |
| Value at Baseline | -- | 0.37 | 0.02 | 0.72 | 0.040 | 0.15 | -0.31 | 0.61 | 0.519 |
| Value at completion of Paclitaxel | -- | Not included in the analysis | | | | 0.48 | 0.17 | 0.80 | 0.003 |
| Luminal status | Luminal A | Reference | | | | Reference | | | |
|  | Luminal B | 0.10 | -0.14 | 0.34 | 0.425 | 0.27 | -0.04 | 0.58 | 0.092 |
|  | Non-Luminal | 0.34 | -0.03 | 0.72 | 0.076 | 0.15 | -0.33 | 0.63 | 0.547 |
| Highest Haemoglobin | -- | 0.04 | -0.03 | 0.11 | 0.307 | 0.04 | -0.04 | 0.13 | 0.336 |
| Albumin | -- | 0.14 | -0.10 | 0.38 | 0.265 | 0.18 | -0.12 | 0.49 | 0.249 |
| **Sural Amplitude** | | | | | | | | | |
| Treatment | Group A | 6.20 | 3.34 | 9.05 | <0.001 | 3.42 | -1.29 | 8.12 | 0.160 |
|  | Group B | Reference | | | | Reference | | | |
| Value at Baseline | -- | 0.27 | 0.14 | 0.39 | <0.001 | 0.05 | -0.16 | 0.26 | 0.635 |
| Value at completion of Paclitaxel | -- | Not included in the analysis | | | | 0.48 | 0.12 | 0.84 | 0.011 |
| Luminal status | Luminal A | Reference | | | | Reference | | | |
|  | Luminal B | -0.46 | -4.82 | 3.89 | 0.835 | -2.68 | -9.01 | 3.65 | 0.410 |
|  | Non-Luminal | -2.67 | -9.33 | 3.98 | 0.434 | -2.38 | -12.10 | 7.33 | 0.633 |
| Highest Haemoglobin | -- | -1.43 | -2.66 | -0.20 | 0.026 | 0.02 | -1.84 | 1.88 | 0.981 |
| Albumin | -- | 2.02 | -2.26 | 6.30 | 0.358 | -2.72 | -8.98 | 3.53 | 0.397 |
| **Sural Velocity** | | | | | | | | | |
| Treatment | Group A | 3.70 | 1.07 | 6.34 | 0.008 | 1.47 | -2.15 | 5.09 | 0.429 |
|  | Group B | Reference | | | | Reference | | | |
| Value at Baseline | -- | 0.45 | -0.03 | 0.92 | 0.070 | -0.13 | -0.76 | 0.50 | 0.692 |
| Value at completion of Paclitaxel | -- | Not included in the analysis | | | | 0.47 | 0.15 | 0.79 | 0.006 |
| Luminal status | Luminal A | Reference | | | | Reference | | | |
|  | Luminal B | 0.12 | -3.92 | 4.16 | 0.954 | -2.15 | -7.39 | 3.08 | 0.423 |
|  | Non-Luminal | -1.81 | -7.91 | 4.30 | 0.564 | -0.34 | -8.28 | 7.60 | 0.933 |
| Highest Haemoglobin | -- | -0.50 | -1.71 | 0.71 | 0.420 | 0.29 | -1.28 | 1.87 | 0.717 |
| Albumin | -- | -2.73 | -6.68 | 1.22 | 0.180 | -1.91 | -7.10 | 3.28 | 0.474 |

CI: Confidence interval; LL – Lower Limit; UL – Upper Limit

^*^Adjusted for Molecular phenotype (Luminal A / Luminal B/ Non-Luminal), Highest recorded haemoglobin, serum albumin and baseline value

^#^Adjusted for Molecular phenotype (Luminal A / Luminal B/ Non-Luminal), Highest recorded haemoglobin, serum albumin, baseline value and value at the time of completion of paclitaxel therapy

**Supplementary Table – 7:** **Multivariable analysis of Quality-of-Life measures (Group A – Interventional group and Group B – Control group)**

| **Variables** | **Levels** | **Dependent Variable: Value at the time of completion of paclitaxel therapy^*^** | | | | **Dependent Variable: Value at 3 times post completion of paclitaxel therapy^#^** | | | |
| --- | --- | --- | --- | --- | --- | --- | --- | --- | --- |
|  |  | **Estimate** | **95% CI LL** | **95% CI UL** | **P-value** | **Estimate** | **95% CI LL** | **95% CI UL** | **P-value** |
| **European Organization for Research and Treatment of Cancer Quality of Life - Sensory** | | | | | | | | | |
| Treatment | Group A | -5.46 | -5.83 | -5.09 | <0.001 | -1.99 | -3.15 | -0.83 | 0.001 |
|  | Group B | Reference | | | | Reference | | | |
| Value at Baseline | -- | Not included in the analysis | | | | Not included in the analysis | | | |
| Value at completion of Paclitaxel | -- | Not included in the analysis | | | | 0.75 | 0.55 | 0.96 | <0.001 |
| Luminal status | Luminal A | Reference | | | | Reference | | | |
|  | Luminal B | 0.80 | 0.23 | 1.37 | 0.007 | 0.29 | -0.22 | 0.79 | 0.270 |
|  | Non-Luminal | 0.60 | -0.26 | 1.46 | 0.179 | 0.40 | -0.33 | 1.13 | 0.291 |
| Highest Haemoglobin | -- | 0.01 | -0.15 | 0.17 | 0.870 | 0.02 | -0.11 | 0.16 | 0.728 |
| Albumin | -- | -0.25 | -0.81 | 0.30 | 0.375 | 0.10 | -0.36 | 0.57 | 0.669 |
| **European Organization for Research and Treatment of Cancer Quality of Life - Motor** | | | | | | | | | |
| Treatment | Group A | -3.82 | -4.32 | -3.33 | <0.001 | -4.20 | -4.71 | -3.68 | <0.001 |
|  | Group B | Reference | | | | Reference | | | |
| Value at Baseline | -- | Not included in the analysis | | | | Not included in the analysis | | | |
| Value at completion of Paclitaxel | -- | Not included in the analysis | | | | 0.58 | 0.46 | 0.70 | <0.001 |
| Luminal status | Luminal A | Reference | | | | Reference | | | |
|  | Luminal B | 0.48 | -0.29 | 1.25 | 0.225 | 0.02 | -0.36 | 0.39 | 0.937 |
|  | Non-Luminal | -0.01 | -1.17 | 1.15 | 0.988 | 0.24 | -0.32 | 0.81 | 0.398 |
| Highest Haemoglobin | -- | 0.01 | -0.21 | 0.22 | 0.953 | -0.11 | -0.21 | <0.01 | 0.047 |
| Albumin | -- | -0.22 | -0.97 | 0.53 | 0.570 | 0.17 | -0.19 | 0.54 | 0.352 |
| **European Organization for Research and Treatment of Cancer Quality of Life – Autonomic** | | | | | | | | | |
| Treatment | Group A | <0.01 | <0.01 | <0.01 | 0.399 | <0.01 | <0.01 | <0.01 | 0.399 |
|  | Group B | Reference | | | | Reference | | | |
| Value at Baseline | -- | Not included in the analysis | | | | Not included in the analysis | | | |
| Value at completion of Paclitaxel | -- | Not included in the analysis | | | | Not included in the analysis | | | |
| Luminal status | Luminal A | Reference | | | | Reference | | | |
|  | Luminal B | <0.01 | <0.01 | <0.01 | 0.694 | <0.01 | <0.01 | <0.01 | 0.694 |
|  | Non-Luminal | <0.01 | <0.01 | <0.01 | 0.816 | <0.01 | <0.01 | <0.01 | 0.816 |
| Highest Haemoglobin | -- | <0.01 | <0.01 | <0.01 | 0.926 | <0.01 | <0.01 | <0.01 | 0.926 |
| Albumin | -- | <0.01 | <0.01 | <0.01 | 0.827 | <0.01 | <0.01 | <0.01 | 0.827 |
| **European Organization for Research and Treatment of Cancer Quality of Life - Overall** | | | | | | | | | |
| Treatment | Group A | -9.29 | -9.93 | -8.64 | <0.001 | -6.64 | -8.03 | -5.24 | <0.001 |
|  | Group B | Reference | | | | Reference | | | |
| Value at Baseline | -- | Not included in the analysis | | | | Not included in the analysis | | | |
| Value at completion of Paclitaxel | -- | Not included in the analysis | | | | 0.63 | 0.49 | 0.78 | <0.001 |
| Luminal status | Luminal A | Reference | | | | Reference | | | |
|  | Luminal B | 1.28 | 0.30 | 2.27 | 0.013 | 0.37 | -0.24 | 0.98 | 0.238 |
|  | Non-Luminal | 0.59 | -0.91 | 2.08 | 0.444 | 0.71 | -0.17 | 1.60 | 0.120 |
| Highest Haemoglobin | -- | 0.02 | -0.26 | 0.30 | 0.889 | -0.08 | -0.25 | 0.08 | 0.323 |
| Albumin | -- | -0.47 | -1.44 | 0.49 | 0.342 | 0.26 | -0.32 | 0.83 | 0.381 |
| **Fullerton Advanced Balance Scale** | | | | | | | | | |
| Treatment | Group A | 1.31 | 0.91 | 1.70 | <0.001 | <0.01 | <0.01 | <0.01 | 0.446 |
|  | Group B | Reference | | | | Reference | | | |
| Value at Baseline | -- | 0.71 | 0.64 | 0.78 | <0.001 | 1.00 | 1.00 | 1.00 | <0.001 |
| Value at completion of Paclitaxel | -- | Not included in the analysis | | | | <0.01 | <0.01 | <0.01 | 0.169 |
| Luminal status | Luminal A | Reference | | | | Reference | | | |
|  | Luminal B | -0.71 | -1.32 | -0.10 | 0.026 | <0.01 | <0.01 | <0.01 | 0.988 |
|  | Non-Luminal | -0.04 | -0.96 | 0.88 | 0.934 | <0.01 | <0.01 | <0.01 | 0.973 |
| Highest Haemoglobin | -- | -0.09 | -0.26 | 0.08 | 0.303 | <0.01 | <0.01 | <0.01 | 0.743 |
| Albumin | -- | -0.20 | -0.80 | 0.39 | 0.504 | <0.01 | <0.01 | <0.01 | 0.791 |
| **Functional Assessment of Cancer Therapy–Taxane: Physical Well-Being** | | | | | | | | | |
| Treatment | Group A | 0.51 | 0.31 | 0.71 | <0.001 | <0.01 | <0.01 | <0.01 | <0.001 |
|  | Group B | Reference | | | | Reference | | | |
| Value at Baseline | -- | -0.02 | -0.09 | 0.05 | 0.539 | <0.01 | <0.01 | <0.01 | <0.001 |
| Value at completion of Paclitaxel | -- | Not included in the analysis | | | | 1.00 | 1.00 | 1.00 | <0.001 |
| Luminal status | Luminal A | Reference | | | | Reference | | | |
|  | Luminal B | 0.01 | -0.27 | 0.30 | 0.920 | <0.01 | <0.01 | <0.01 | <0.001 |
|  | Non-Luminal | 0.25 | -0.18 | 0.68 | 0.257 | <0.01 | <0.01 | <0.01 | <0.001 |
| Highest Haemoglobin | -- | -0.02 | -0.10 | 0.06 | 0.568 | <0.01 | <0.01 | <0.01 | <0.001 |
| Albumin | -- | 0.26 | -0.01 | 0.54 | 0.065 | <0.01 | <0.01 | <0.01 | <0.001 |
| **Functional Assessment of Cancer Therapy–Taxane: Social/ Family Well-Being** | | | | | | | | | |
| Treatment | Group A | -1.85 | -2.49 | -1.21 | <0.001 | <0.01 | <0.01 | <0.01 | <0.001 |
|  | Group B | Reference | | | | Reference | | | |
| Value at Baseline | -- | -0.19 | -0.41 | 0.03 | 0.091 | <0.01 | <0.01 | <0.01 | 0.100 |
| Value at completion of Paclitaxel | -- | Not included in the analysis | | | | 1.00 | 1.00 | 1.00 | <0.001 |
| Luminal status | Luminal A | Reference | | | | Reference | | | |
|  | Luminal B | 0.15 | -0.83 | 1.13 | 0.766 | <0.01 | <0.01 | <0.01 | 0.682 |
|  | Non-Luminal | -0.37 | -1.85 | 1.11 | 0.626 | <0.01 | <0.01 | <0.01 | 0.892 |
| Highest Haemoglobin | -- | -0.02 | -0.30 | 0.26 | 0.885 | <0.01 | <0.01 | <0.01 | 0.511 |
| Albumin | -- | -0.91 | -1.86 | 0.04 | 0.066 | <0.01 | <0.01 | <0.01 | 0.190 |
| **Functional Assessment of Cancer Therapy–Taxane: Emotional Well-being** | | | | | | | | | |
| Treatment | Group A | <0.01 | <0.01 | <0.01 | 0.190 | <0.01 | <0.01 | <0.01 | 0.190 |
|  | Group B | Reference | | | | Reference | | | |
| Value at Baseline | -- | <0.01 | <0.01 | <0.01 | 0.072 | <0.01 | <0.01 | <0.01 | 0.072 |
| Value at completion of Paclitaxel | -- | Not included in the analysis | | | | Not included in the analysis | | | |
| Luminal status | Luminal A | Reference | | | | Reference | | | |
|  | Luminal B | <0.01 | <0.01 | <0.01 | 0.631 | <0.01 | <0.01 | <0.01 | 0.631 |
|  | Non-Luminal | <0.01 | <0.01 | <0.01 | 0.962 | <0.01 | <0.01 | <0.01 | 0.962 |
| Highest Haemoglobin | -- | <0.01 | <0.01 | <0.01 | 0.972 | <0.01 | <0.01 | <0.01 | 0.972 |
| Albumin | -- | <0.01 | <0.01 | <0.01 | 0.807 | <0.01 | <0.01 | <0.01 | 0.807 |
| **Functional Assessment of Cancer Therapy–Taxane: Functional Well-Being** | | | | | | | | | |
| Treatment | Group A | -0.02 | -0.57 | 0.54 | 0.953 | <0.01 | <0.01 | <0.01 | <0.001 |
|  | Group B | Reference | | | | Reference | | | |
| Value at Baseline | -- | 0.07 | <0.01 | 0.15 | 0.049 | <0.01 | <0.01 | <0.01 | <0.001 |
| Value at completion of Paclitaxel | -- |  | | | | 1.00 | 1.00 | 1.00 | <0.001 |
| Luminal status | Luminal A | Reference | | | | Reference | | | |
|  | Luminal B | -0.79 | -1.64 | 0.06 | 0.073 | <0.01 | <0.01 | <0.01 | <0.001 |
|  | Non-Luminal | -1.68 | -2.98 | -0.39 | 0.013 | <0.01 | <0.01 | <0.01 | <0.001 |
| Highest Haemoglobin | -- | -0.07 | -0.31 | 0.18 | 0.594 | <0.01 | <0.01 | <0.01 | <0.001 |
| Albumin | -- | 0.03 | -0.80 | 0.87 | 0.943 | <0.01 | <0.01 | <0.01 | <0.001 |
| **Functional Assessment of Cancer Therapy–Taxane: Taxane-Specific Subscale (TAX-S)** | | | | | | | | | |
| Treatment | Group A | -1.08 | -4.42 | 2.26 | 0.530 | <0.01 | <0.01 | <0.01 | 0.982 |
|  | Group B | Reference | | | | Reference | | | |
| Value at Baseline | -- | 1.53 | -0.79 | 3.85 | 0.201 | <0.01 | <0.01 | <0.01 | 0.467 |
| Value at completion of Paclitaxel | -- |  | | | | 1.00 | 1.00 | 1.00 | <0.001 |
| Luminal status | Luminal A | Reference | | | | Reference | | | |
|  | Luminal B | 4.75 | -0.33 | 9.84 | 0.072 | <0.01 | <0.01 | <0.01 | 0.891 |
|  | Non-Luminal | 8.11 | 0.35 | 15.86 | 0.045 | <0.01 | <0.01 | <0.01 | 0.991 |
| Highest Haemoglobin | -- | 0.90 | -0.54 | 2.35 | 0.223 | <0.01 | <0.01 | <0.01 | 0.531 |
| Albumin | -- | -1.74 | -6.72 | 3.24 | 0.496 | <0.01 | <0.01 | <0.01 | 0.545 |
| **Functional Assessment of Cancer Therapy–Taxane: Trial Outcome Index** | | | | | | | | | |
| Treatment | Group A | -1.03 | -3.93 | 1.88 | 0.492 | <0.01 | <0.01 | <0.01 | 0.967 |
|  | Group B | Reference | | | | Reference | | | |
| Value at Baseline | -- | -0.41 | -0.80 | -0.03 | 0.038 | <0.01 | <0.01 | <0.01 | 0.464 |
| Value at completion of Paclitaxel | -- |  | | | | 1.00 | 1.00 | 1.00 | <0.001 |
| Luminal status | Luminal A | Reference | | | | Reference | | | |
|  | Luminal B | 3.70 | -0.65 | 8.05 | 0.100 | <0.01 | <0.01 | <0.01 | 0.956 |
|  | Non-Luminal | 7.02 | 0.40 | 13.65 | 0.042 | <0.01 | <0.01 | <0.01 | 0.930 |
| Highest Haemoglobin | -- | 0.74 | -0.49 | 1.98 | 0.241 | <0.01 | <0.01 | <0.01 | 0.645 |
| Albumin | -- | -1.87 | -6.15 | 2.42 | 0.396 | <0.01 | <0.01 | <0.01 | 0.723 |
| **Functional Assessment of Cancer Therapy–Taxane: General (FACT-G)** | | | | | | | | | |
| Treatment | Group A | -1.28 | -2.11 | -0.44 | 0.004 | <0.01 | <0.01 | <0.01 | <0.001 |
|  | Group B | Reference | | | | Reference | | | |
| Value at Baseline | -- | 0.03 | -0.05 | 0.12 | 0.463 | <0.01 | <0.01 | <0.01 | <0.001 |
| Value at completion of Paclitaxel | -- |  | | | | 1.00 | 1.00 | 1.00 | <0.001 |
| Luminal status | Luminal A | Reference | | | | Reference | | | |
|  | Luminal B | -0.63 | -1.89 | 0.63 | 0.331 | <0.01 | <0.01 | <0.01 | <0.001 |
|  | Non-Luminal | -1.67 | -3.59 | 0.25 | 0.094 | <0.01 | <0.01 | <0.01 | <0.001 |
| Highest Haemoglobin | -- | -0.08 | -0.44 | 0.28 | 0.665 | <0.01 | <0.01 | <0.01 | <0.001 |
| Albumin | -- | -0.60 | -1.85 | 0.64 | 0.343 | <0.01 | <0.01 | <0.01 | <0.001 |
| **Functional Assessment of Cancer Therapy–Taxane: Total (FACT-Total)** | | | | | | | | | |
| Treatment | Group A | -2.59 | -5.51 | 0.34 | 0.088 | <0.01 | <0.01 | <0.01 | <0.001 |
|  | Group B | Reference | | | | Reference | | | |
| Value at Baseline | -- | -0.30 | -0.65 | 0.04 | 0.093 | <0.01 | <0.01 | <0.01 | <0.001 |
| Value at completion of Paclitaxel | -- |  | | | | 1.00 | 1.00 | 1.00 | <0.001 |
| Luminal status | Luminal A | Reference | | | | Reference | | | |
|  | Luminal B | 3.87 | -0.54 | 8.28 | 0.091 | <0.01 | <0.01 | <0.01 | <0.001 |
|  | Non-Luminal | 6.58 | -0.14 | 13.29 | 0.059 | <0.01 | <0.01 | <0.01 | <0.001 |
| Highest Haemoglobin | -- | 0.73 | -0.53 | 1.99 | 0.261 | <0.01 | <0.01 | <0.01 | <0.001 |
| Albumin | -- | -2.62 | -6.95 | 1.72 | 0.241 | <0.01 | <0.01 | <0.01 | <0.001 |

CI: Confidence interval; LL – Lower Limit; UL – Upper Limit; EORTC European Organization for Research and Treatment of Cancer Quality of Life

^*^Adjusted for Molecular phenotype (Luminal A / Luminal B/ Non-Luminal), Highest recorded hemoglobin, serum albumin and baseline value (Baseline value not included in the EORTC assessments alone as they were exactly the same value across both the groups)

^#^Adjusted for Molecular phenotype (Luminal A / Luminal B/ Non-Luminal), Highest recorded hemoglobin, serum albumin, baseline value and value at the time of completion of paclitaxel therapy
